# Immune Checkpoint PD-1/PD-L1 CTLA-4/CD80 are Blocked by *Rhus verniciflua* Stokes and its Active Compounds

**DOI:** 10.3390/molecules24224062

**Published:** 2019-11-09

**Authors:** Wei Li, Tae In Kim, Ji Hye Kim, Hwan-Suck Chung

**Affiliations:** Korean Medicine (KM)-Application Center, Korea Institute of Oriental Medicine (KIOM), Daegu 41062, Korea; liwei1986@kiom.re.kr (W.L.); tikim@kiom.re.kr (T.I.K.); jkim2903@kiom.re.kr (J.H.K.)

**Keywords:** *Rhus verniciflua* Stokes, immune checkpoints, PD-1, CTLA-4, flavonoid, polyphenol

## Abstract

The bark of *Rhus verniciflua* Stokes (RVS) has been used to treat cancer in Korean herbal medicine. When we screened for PD-1 and CTLA-4 immune checkpoint inhibitors (PD-1/PD-L1 CTLA-4/CD80) from around 800 herbal extracts using competitive Enzyme-Linked Immunosorbent Assay (ELISA), we found that RVS blocked both the PD-1/PD-L1 and the CTLA-4/CD80 interactions. To identify the active compounds from RVS, we performed bioactivity-guided fractionation, and the ethyl acetate (EtOAc) fraction of RVS proved to be the most effective at blocking the PD-1/PD-L1 and CTLA-4/CD80 interactions. In addition, we isolated and identified 20 major compounds in the EtOAc fraction of RVS and then examined the blocking effects of these 20 compounds on PD-1/PD-L1 and CTLA-4/CD80. Among them, four compounds [eriodictyol (**7**) > fisetin (**9**) > quercetin (**18**) > liquiritigenin (**13**)] blocked the interaction of PD-1/PD-L1 on competitive ELISA. In addition, four different compounds [protocatechuic acid (**2**) > caffeic acid (**19**) > taxifolin (**5**) > butin (**6**)] blocked the interaction of CTLA-4/CD80. Our findings suggest that RVS and its components could be used as a potential immune checkpoint inhibitor blockade and could be developed for immuno-oncological therapeutics.

## 1. Introduction

*Rhus verniciflua* Stokes (RVS) (Anacardiaceae), commonly known as Chinese lacquer tree, is distributed in Korea, Japan, and China [1]. RVS tissues, particularly the bark, have been shown to contain a large number of bioactive phytochemical constituents, including alkaloids, polyphenols, and flavonoids [2,3]. Since ancient times, RVS have been used as herbal medicinal plant to treat various conditions, such as gastroenteritis, arthritis, hypertension, diabetes, stroke, and chronic fatigue disease [3]. However, the blocking effects of this plant on the immune checkpoint inhibitors, such as PD-1/PD-L1 and CTLA-4/CD80, are not currently understood. In the present study, as part of an investigation of novel bioactive constituents in RVS, bioactivity-guided fractionation, and isolation from RVS bark revealed 20 secondary metabolites (**1**–**20**).

Immune checkpoints, which can stimulate or inhibit T cell responses, were well known, as a result of the award of the Nobel Prize in Physiology or Medicine in 2018 to James Allison and Tasuku Honjo for their discovery of CTLA-4 and PD-1, respectively. When CD80 molecules on antigen-presenting cells (APC) interact with CD28 on T cells, T cell activities are stimulated and sustained, whereas when CD80 molecules bind with CTLA-4, a negative signal is sent to activated T cells [4]. Similarly, T cell proliferation and cytokine production were inhibited when PD-1 on T cells interacted with PD-L1 or PD-L2 on APC or tumor cells [5]. Blocking monoclonal antibodies for PD-1 (Pembrolizumab, Nivolumab, and Cemiplimab), PD-L1 (Atezolizumab, Avelumab, and Durvalumab), and CTLA-4 (Ipilimumab) have been approved by the US Food and Drug Administration and have been used for treatment of metastatic melanoma and non-small lung cancer [6]. However, there have been many cases of immune-related adverse events such as colitis, thyroiditis and type 1 diabetes in response to these monoclonal antibodies [7]. In addition, these monoclonal antibodies are expensive and show limited impact on solid tumors because antibodies are large molecules cannot easily penetrate such a tumor. Various studies using small molecules to overcome the limitation of monoclonal antibody therapy have been conducted recently [8,9], but most of these studies have not succeeded because of low effectiveness as well as toxicities associated with these drugs. However, oriental herbal medicines, which have a long anecdotal history of safe use, are promising anticancer drug candidates because their toxicities and side effects are well known. In the present study, we screened approximately 800 herbal medicines for their potential blocking effects on PD-1/PD-L1 and CTLA-4/CD80, and discovered that RVS blocked both the immune checkpoint inhibitors PD-1/PD-L1 and CTLA-4/CD80 in competitive Enzyme-Linked Immunosorbent Assay (ELISA) studies.

## 2. Results

### 2.1. RVS Blocks the PD-1/PD-L1 Interaction

**Figure 1:**
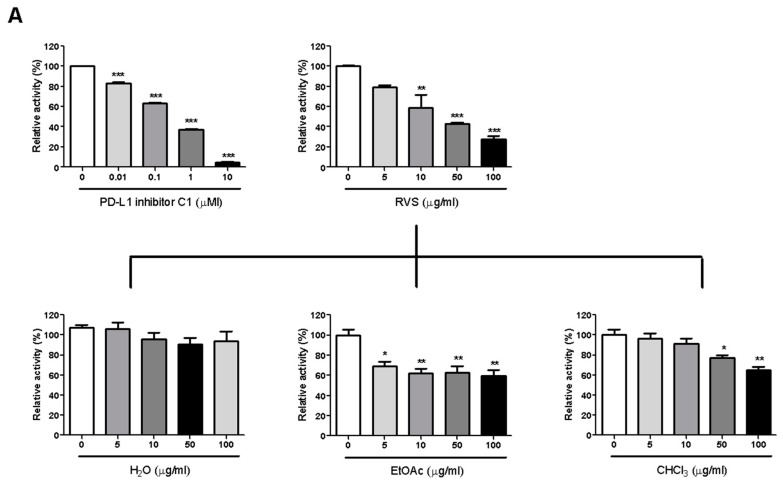

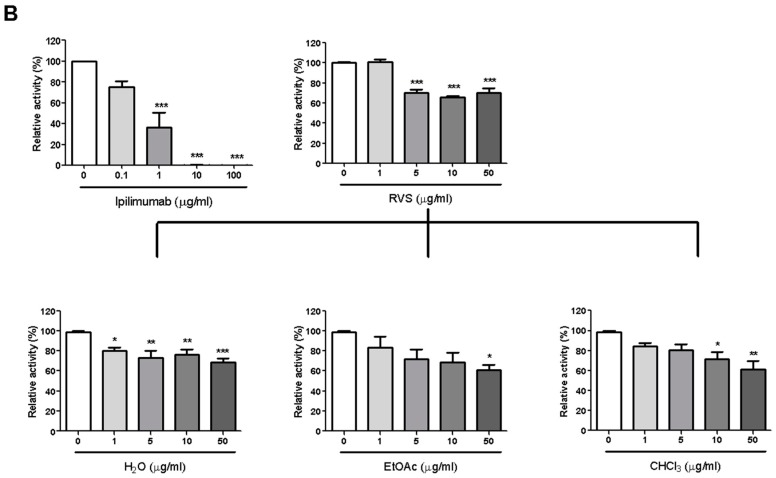
Immune checkpoint blocking effects of *Rhus verniciflua* Stokes (RVS) extract and fractions tested by competitive Enzyme-Linked Immunosorbent Assay (ELISA). Effect of PD-L1 inhibitor C1, RVS extract and fractions on PD-1/PD-L1 binding activity (**A**); Effect of anti-CTLA-4 antibody, RVS extract and fractions on CTLA-4/CD80 binding activity (**B**). The relative binding activity was normalized to the relative percentage of the vehicle control group. Half-maximal inhibitory concentration (IC_50_) was calculated using Prism “log[inhibitor] vs. normalized response” equation. All results are presented as the mean value of three independent biological replicates. * *p* < 0.05, ** *p* < 0.01, *** *p* < 0.001, compared with the vehicle control group.

We investigated PD-1/PD-L1 blocking effect by RVS using competition ELISA. RVS blocked the PD-1/PD-L1 interaction in a dose-dependent manner, with a half-maximal inhibitory concentration (IC_50_) at 26.22 μg/mL. To identify the main constituents of RVS that blocked activity against PD-1/PD-L1 binding, we partitioned the RVS extract with ethyl acetate (EtOAc), chloroform (CHCl_3_) and water (H_2_O). The EtOAc fraction of the extract showed more effective blocking efficacy than did other fractions. This observation indicates that the blocking effect of RVS on the PD-1/PD-L1 interaction was attributable to constituents enriched in the EtOAc fraction (Figure 1A).

### 2.2. RVS Blocks the CTLA-4/CD80 Interaction

The CTLA-4/CD80 blocking activity of RVS was examined via competition ELISA as described before. Similar to the results with respect to the PD-1/PD-L1 blockade, RVS blocked 29.9% of CTLA-4/CD80 interaction at 5μg/mL and the EtOAc fraction exhibited the most effective blocking activity on CTLA-4/CD80 binding (Figure 1B).

### 2.3. Structural Elucidation of Compounds ***1***–***20***

**Figure 2:**
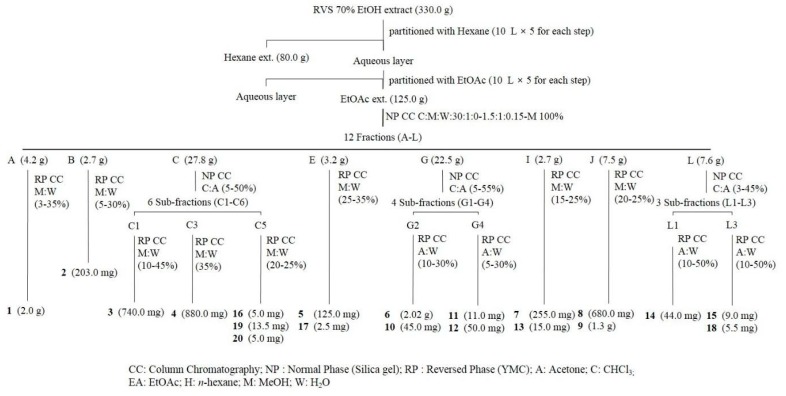
Isolation scheme of compounds **1**–**20** from *Rhus verniciflua* Stokes (RVS).

**Figure 3:**
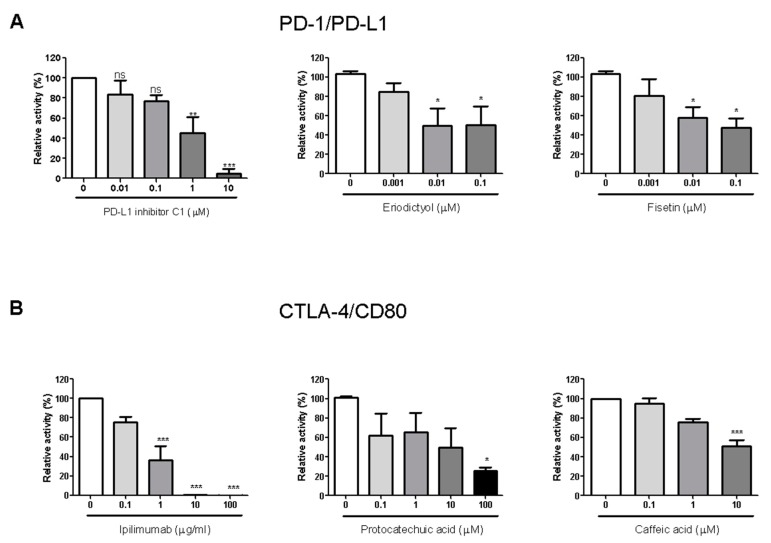
Immune checkpoint blocking effects of isolated compounds by competitive Enzyme-Linked Immunosorbent Assay (ELISA). Effect of PD-L1 inhibitor C1, compounds eriodictyol (**7**) and fisetin on PD-1/PD-L1 binding activity (**9**) (**A**); effect of anti-CTLA-4 antibody (Ipilimumab), compounds protocatechuic acid (**2**) and caffeic acid (**19**) on CTLA-4/CD80 binding activity (**B**). The relative binding activity was normalized to the relative percentage of the vehicle control group. Half-maximal inhibitory concentration (IC_50_) was calculated using Prism “log[inhibitor] vs. normalized response” equation. All results are presented as the mean of three independent biological replicates. * *p* < 0.05, ** *p* < 0.01, *** *p* < 0.001, compared with the vehicle control group.

Using combined chromatographic separations (Figure 2), 20 secondary metabolites were isolated from RVS, including four benzoic acids (**1**–**3** and **19**), 11 flavonoids (**4**–**13** and **18**), and five polyphenols (**14**–**17** and **20**). Their structures were identified as gallic acid (**1**), protocatechuic acid (**2**), gallic acid cetyl ester (**3**), fustin (**4**) [10], taxifolin (**5**) [11], butin (**6**) [11], eriodictyol (**7**) [12], 5-deoxyluteolin (**8**) [3], fisetin (**9**) [13], garbanzol (**10**) [13], aromadendrin (**11**) [14], naringenin (**12**) [14], liquiritigenin (**13**) [14], sulfuretin (**14**) [13], rhusopolyphenol F (**15**) [10], rhusopolyphenol B (**16**) [10], rhusopolyphenol A (**17**) [10], quercetin (**18**) [11], caffeic acid (**19**), and 2-benzyl-2,3′,4′,6-tetrahydroxybenzo[*b*]furan-3(2*H*)-one (**20**) [10] (Figure 3). Their structures were elucidated by 1-D and 2-D nuclear magnetic resonance (NMR), mass spectrometry, and compared with those reported in the literature (Figures S2–S21). In this study, to the best of our knowledge, compounds **11**–**13** were isolated from *R. verniciflua* for the first time.

### 2.4. PD-1/PD-L1 Blocking Effect of Isolated Compounds

The 20 compounds isolated from the EtOAc fraction of RVS were tested for their PD-1/PD-L1 blocking effects (Figure S22). Small molecule PD-L1 inhibitor C1 was used as a positive control (IC_50_ value 0.55 μM). Compounds **7**, **9**, **13**, and **18** showed blocking effects, with IC_50_ values of 0.04, 0.04, 11.85, and 5.71 μM, respectively. Of these, compounds **7** and **9** in particular exhibited strong blocking effects, with IC_50_ values below 1 μM (Figure 3A).

### 2.5. CTLA-4/CD80 Blocking Effect of Isolated Compounds

The 20 compounds isolated from the EtOAc fraction were tested for CTLA-4/CD80 blocking activity (Figure S23). Anti-CTLA-4 antibody was used as a positive control (IC_50_ 0.43 μg/mL). Compounds **2**, **5**, **6**, and **19** showed blocking effect, with IC_50_ values of 4.51, 35.45, 134.10, and 10.04 μM, respectively (Figure 3B).

## 3. Discussion

Many preclinical studies have been carried out on the anticancer effects of RVS [15,16,17,18,19,20,21], including, in two cases, clinical reports of treatment for metastatic renal cell carcinoma [22]. To our knowledge, there has been no report of an immuno-oncological study on RVS. Most of the anticancer studies on RVS have described the apoptotic effects of RVS [23,24,25] and its mechanism of action [26].

Competitive ELISA results showed that RVS markedly blocked both the PD-1/PD-L1 and CTLA-4/CD80 interactions. Further, we identified eriodictyol (**7**) and fisetin (**9**) as being active compounds for blocking the PD-1/PD-L1 interaction, and protocatechuic acid (**2**) and caffeic acid (**19**) as being active compounds for blocking the CTLA-4/CD80 interaction. According to previous studies, some natural compounds, known as Pan-assay interference compounds (PAINS), have nonspecific activity [27,28]. Toxoflavin, isothiazolones, hydroxyphenyl hydrazones, curcumin, phenol-sulfonamides, rhodanines, enones, quianones, and catechols have all been reported as common PAINS [29]. If the RVS compounds active with respect to the blocking of PD-1/PD-L1 are PAINS that react nonspecifically with biological targets, they should nonspecifically block CTLA-4/CD80 as well. But the RVS compounds that actively blocked PD-1/PD-L1 did not block CTLA-4/CD80, and vice versa. Based on these observed results, therefore, we cautiously speculate that the active compounds from RVS may not be PAINS [30].

Recent studies have shown that immune checkpoint blockades have synergistic effects with conventional cancer therapies, such as chemotherapy and radiation therapy [31]. Immune checkpoint blockades are known to stimulate T cell activation, whereas the conventional cancer therapies promote antigen release and presentation [31]. In this respect, RVS, which is known for its anticancer effects like chemotherapy, would kill tumor cells and then promote antigen presentation. In addition, RVS, which can block PD-1/PD-L1 and CTLA-4/CD80 checkpoints, would stimulate the sustained activation of T cells. For the future study, we will examine the immuno-oncological activity of RVS in cell model and animal model systems.

## 4. Materials and Methods

### 4.1. General Experimental Procedures

The NMR spectra were recorded using a Bruker Avance III 600 NMR spectrometer (^1^H, 600 MHz; ^13^C, 150 MHz) (Bruker Biospin GmbH, Karlsruhe, Germany), with tetramethylsilane (TMS) as an internal standard. Heteronuclear multiple quantum correlation, heteronuclear multiple bond correlation, rotating frame nuclear overhauser effect spectroscopy (ROESY), and ^1^H–^1^H correlation spectroscopy (COSY) spectra were recorded using a pulsed-field gradient. The high-resolution–electrospray ionization–mass spectrometry (HR-ESI-MS) spectra were obtained using an Agilent 1200 Liquid Chromatography/Mass Selective Detector (LC/MSD) Trap spectrometer (Agilent, Santa Clara, CA, USA). Preparative-HPLC was performed using a Gilson 321 pump, a 151 UV/VIS detector (Gilson SAS, Villiers-le-Bel, France), and a RS Tech HECTOR-M C18 column (5-µm particle size, 250 × 21.2 mm) (RS Tech Corp, Chungju, South Korea). Column chromatography was performed using silica gel (Kieselgel 60, 70–230, and 230–400 mesh; Merck, Darmstadt, Germany), and YMC C18 resin. Thin-layer chromatography was performed using pre-coated silica gel 60 F_254_ and RP-18 F_254S_ plates (both 0.25-mm thickness; Merck, Darmstadt, Germany), the spots being detected under UV light and using 10% H_2_SO_4_.

### 4.2. Plant Material

Dried bark of RVS was kindly provided from Bomyeong Herbal Market, Seoul in 2018. Its identity was confirmed by one of the authors (Dr. Wei Li). A voucher specimen (IC-180018) was deposited at the Herbarium of Korean Medicine (KM)-Application Center, Korea Institute of Oriental Medicine, Republic of Korea.

### 4.3. Extraction and Isolation of Chemicals

The dried bark of RVS (8.0 kg) was exhaustively extracted under reflux with 70% EtOH three times, each time with 50 L solvent. The total 70% EtOH extract (330.0 g) was suspended in deionized water and partitioned with CHCl_3_ (80.0 g), with the water fraction being partitioned sequentially with EtOAc (125.0 g). The EtOAc fraction was subjected to silica gel column chromatography with a gradient of CHCl_3_-MeOH-H_2_O (30:1:0, 15:1:0, 10:1:0, 6:1:0.1, 5:1:0.1, 4:1:0.1, 3:1:0.1, 1.5:1:0.15, MeOH; 8.0 L for each step) to give 12 fractions (fractions A-L). Fraction A was isolated with a gradient of MeOH-H_2_O (3–35%) by middle-pressure liquid chromatography (MPLC) using a YMC C18 column to give compound **1** (2.0 g). Fraction B was isolated with a gradient of MeOH-H_2_O (5–30%) by MPLC using YMC C18 column to give compound **2** (203.0 mg). Fraction C was separated on a silica gel column (2.5 × 80 cm) with a gradient of CHCl_3_-acetone (5–50%) to give six sub-fractions (C1–C6). Fraction C1 was isolated by preparative-HPLC (MeOH-H_2_O: 5–30%) to give compound **3** (740.0 mg). Fraction C3 was isolated by preparative-HPLC (MeOH-H_2_O: 35%) to give compound **4** (880.0 mg). Fraction C5 was isolated by preparative-HPLC (MeOH-H_2_O: 20–25%) to give compounds **16** (5.0 mg), **19** (13.5 mg), and **20** (5.0 mg). Fraction E was isolated with a gradient of MeOH-H_2_O (5–30%) by MPLC using a YMC C18 column to give compounds **5** (125.0 mg) and **17** (2.5 mg). Fraction G was separated on a silica gel column (3.0 × 80 cm) with a gradient of CHCl_3_-acetone (5–55%) to give four sub-fractions (G1–G4). Fraction G2 was isolated by preparative-HPLC (acetone-H_2_O: 10–30%) to give compounds **6** (2.02 g) and **10** (45.0 mg). Fraction G4 was isolated by preparative-HPLC (acetone-H_2_O: 5–30%) to give compounds **11** (11.0 mg) and **12** (50.0 mg). Fraction I was isolated with a gradient of MeOH-H_2_O (15–25%) by MPLC using a YMC C18 column to give compounds **7** (255.0 mg) and **13** (15.0 mg). Fraction J was isolated with a gradient of MeOH-H_2_O (20–25%) by MPLC using a YMC C18 column to give compounds **8** (680.0 mg) and **9** (1.3 g). Fraction L was separated on a silica gel column (1.5 × 80 cm) with a gradient of CHCl_3_-acetone (3–45%) to give three sub-fractions (L1-L3). Fraction L1 was isolated by preparative-HPLC (acetone-H_2_O: 10F50%) to give compound **14** (44.0 mg). Fraction L3 was isolated by preparative-HPLC (acetone-H_2_O: 10–50%) to give compounds **15** (9.0 mg) and **18** (5.5 mg).

### 4.4. Chemicals and Antibodies

PD–1/PD-L1 Inhibitor Screening Assay Kit (#72005), anti-PD–1 Neutralizing Antibody (#71120), and CTLA-4/CD80 Inhibitor Screening Assay Kit (#72009) were purchased from BPS Bioscience (San Diego, CA, USA). Anti-CTLA-4 antibody (#A2001) was purchased from Selleck (Houston, TX, USA).

### 4.5. Competitive ELISA

Competitive ELSIA was performed using a PD–1/PD-L1 or CTLA-4/CD80 Inhibitor Screening Assay Kit, according to the supplier’s instructions. An aliquot of 1 μg/mL recombinant human PD-L1 (BPS Bioscience, #71104) or CTLA-4 (BPS Bioscience, #71149) were coated onto a 96-well plate overnight. Plates were washed with phosphate-buffered saline (PBS) containing 0.1% Tween (PBS-T), blocked for 1 h at room temperature with PBS-T containing 2% (*w*/*v*) bovine serum albumen, and washed again. After washing, vehicle or test samples were added, and the reaction continued for 1 h. As a positive control, PD-L1 inhibitor C1 or anti-CTLA-4 neutralizing antibody were used. Biotinylated hPD–1 (BPS Bioscience, #71109) or biotinylated hCD80 (BPS Bioscience, #71114) was added to each well, and plates were incubated for 2 h at room temperature. After three washes with PBS-T, diluted streptavidin-horseradish peroxidase (HRP) was added to each well, and plates were reacted for 1 h while shaking at low speed. After the reaction, plates were washed three times with PBS-T, and HRP substrates A and B were added. Relative chemiluminescence was measured on a SpectraMax L Luminometer (Molecular Devices, San Jose, California, USA) and expressed as the relative binding activity. The results were normalized to the relative percentage of the vehicle control group. Half-maximal inhibitory concentration (IC_50_) was calculated using Prism “log[inhibitor] vs. normalized response” equation. All results are presented as the mean of three independent biological replicates. * *p* < 0.05, ** *p* < 0.01, *** *p* < 0.001, compared with the vehicle control group [32,33,34,35,36,37].

### 4.6. Statistical Analysis

All values are expressed as means ± standard error of the mean. The statistical significance threshold (*p* < 0.05 for all analyses) was assessed by one-way ANOVA followed by Tukey’s post-hoc test for multiple comparisons using Prism 5.01 software (GraphPad Software Inc., San Diego, CA, USA).

## 5. Conclusions

We screened diverse medicinal plants to discover new candidates for immune checkpoint inhibitors. Among them, RVS showed remarkable blocking effect against both PD-1/PD-L1 and CTLA-4/CD80 interaction. To determine active constituents of RVS, we conducted bioactivity-guided fractionation and repeated column chromatography. Also, 20 compounds were isolated from EtOAc fraction of RVS and active compounds such as eriodictyol (**7**) and fisetin (**9**) were identified as inhibitors of PD-1/PD-L1 and protocatechuic acid (**2**) for CTLA-4/CD80 inhibitors. Our findings show that RVS and its active compounds could be applied as candidates of small-molecule inhibitors PD-1 and CTLA-4 immune checkpoint.

## Supplementary Materials

Supplementary materials are available online.
