# Supplementary material for: Immune Checkpoint PD-1/PD-L1 CTLA-4/CD80 are Blocked by Rhus verniciflua Stokes and its Active Compounds
Source: Molecules. 2019 Nov 9;24(22):4062. doi: 10.3390/molecules24224062 (PMC6891444; doi:10.3390/molecules24224062)
Supplement: Supplementary file 1 [file molecules-24-04062-s001.pdf]

Supplementary Material

Immune Checkpoint PD-1/PD-L1 CTLA-4/CD80 are Blocked by *Rhus verniciflua* Stokes and its Active Compounds

Wei Li,[1,#] Tae In Kim,[1,#] Ji Hye Kim,[1] and Hwan-Suck Chung [1],*

[1] Korean medicine (KM)-Application Center, Korea Institute of Oriental Medicine (KIOM), Daegu 41062, Republic of Korea; hschung@kiom.re.kr (H.S.C); liwei1986@kiom.re.kr (W.L.) ; tikim@kiom.re.kr (T.I.K) ; jkim2903@kiom.re.kr (J.H.K)

[#] These authors contributed equally to this work.

***** Correspondence: hschung@kiom.re.kr (H.S.C); Tel: (+82) 53 940 3875; Fax: (+82) 53 940 3899

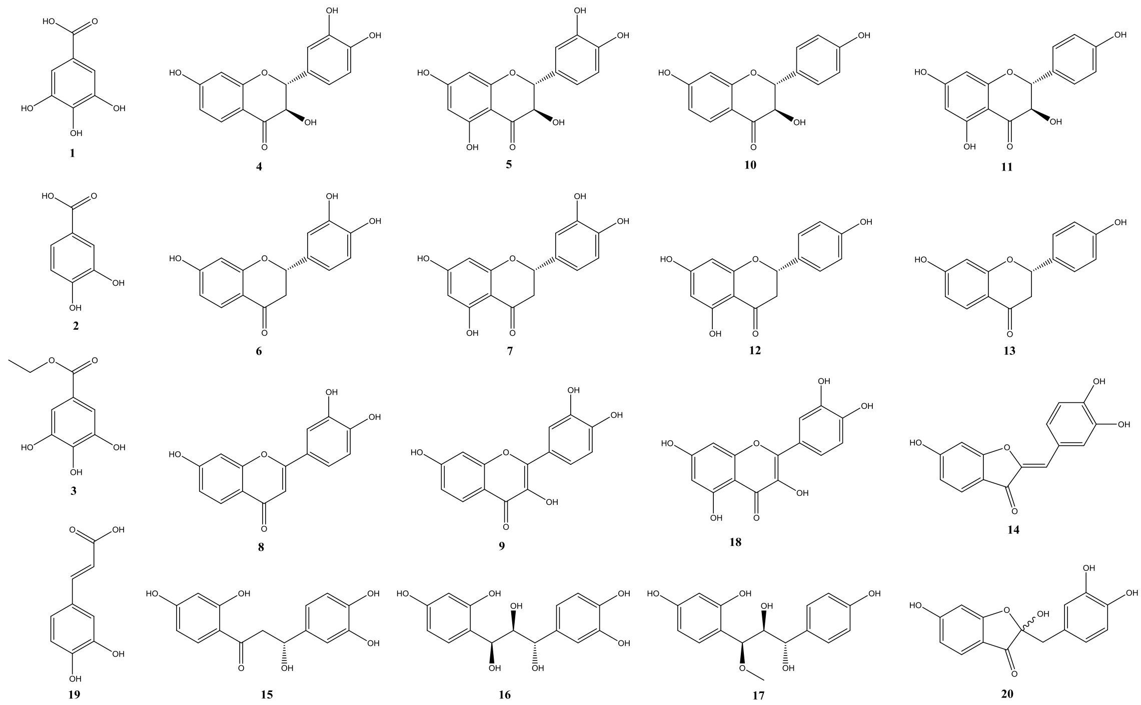

S1. Structures of compounds **1**–**20** from RVS.

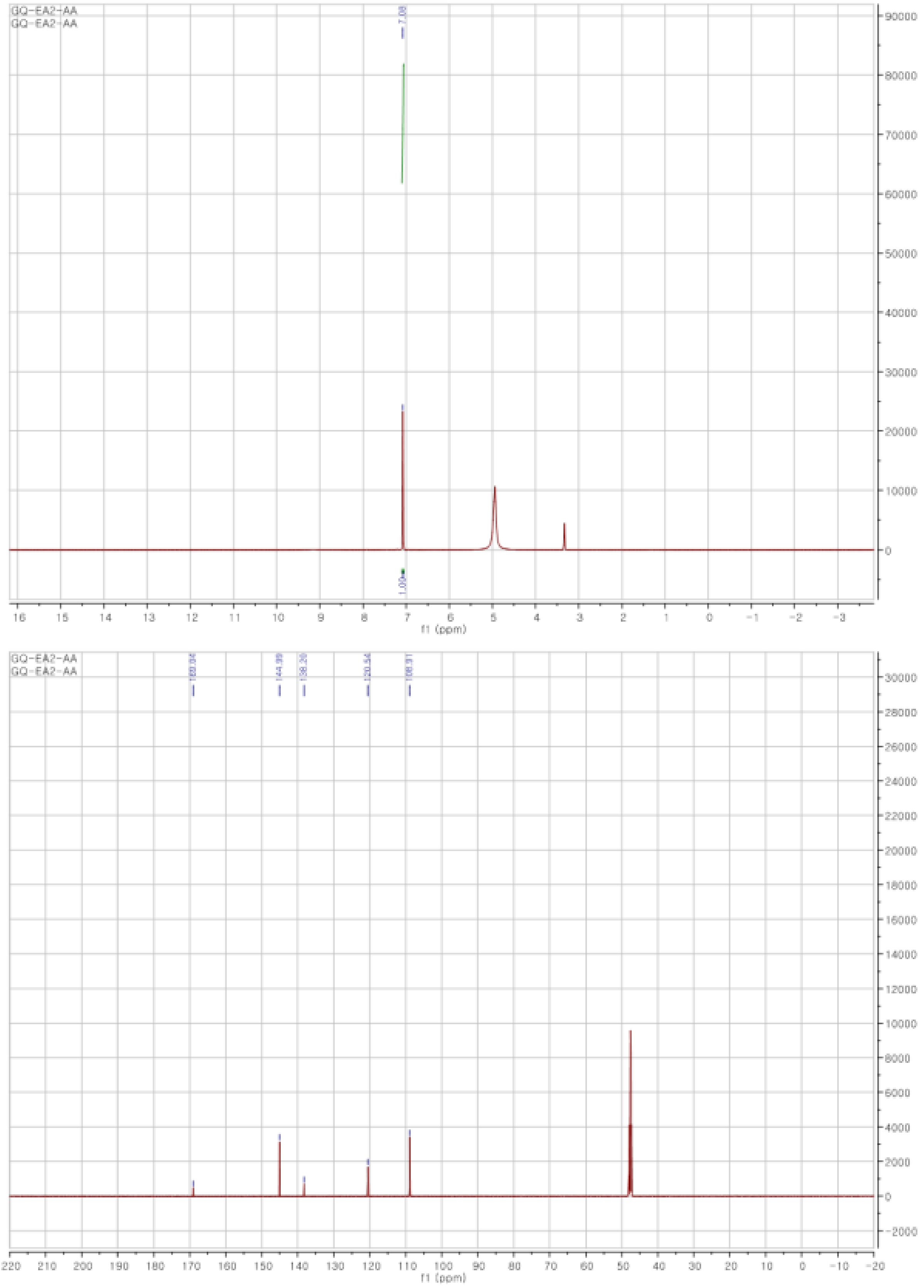

S2. ^{1}H and ^{13}C NMR spectrum of compound **1** in methanol-d_4 (600 and 150 MHz)

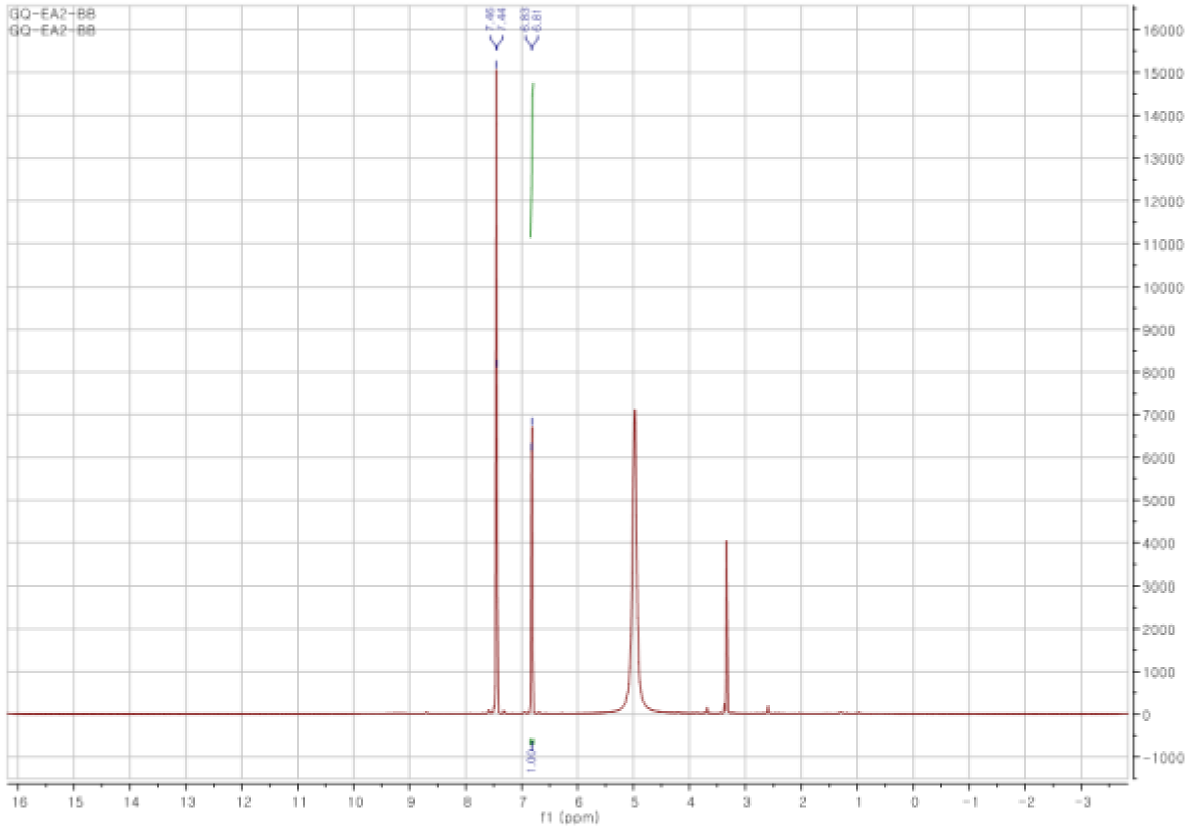

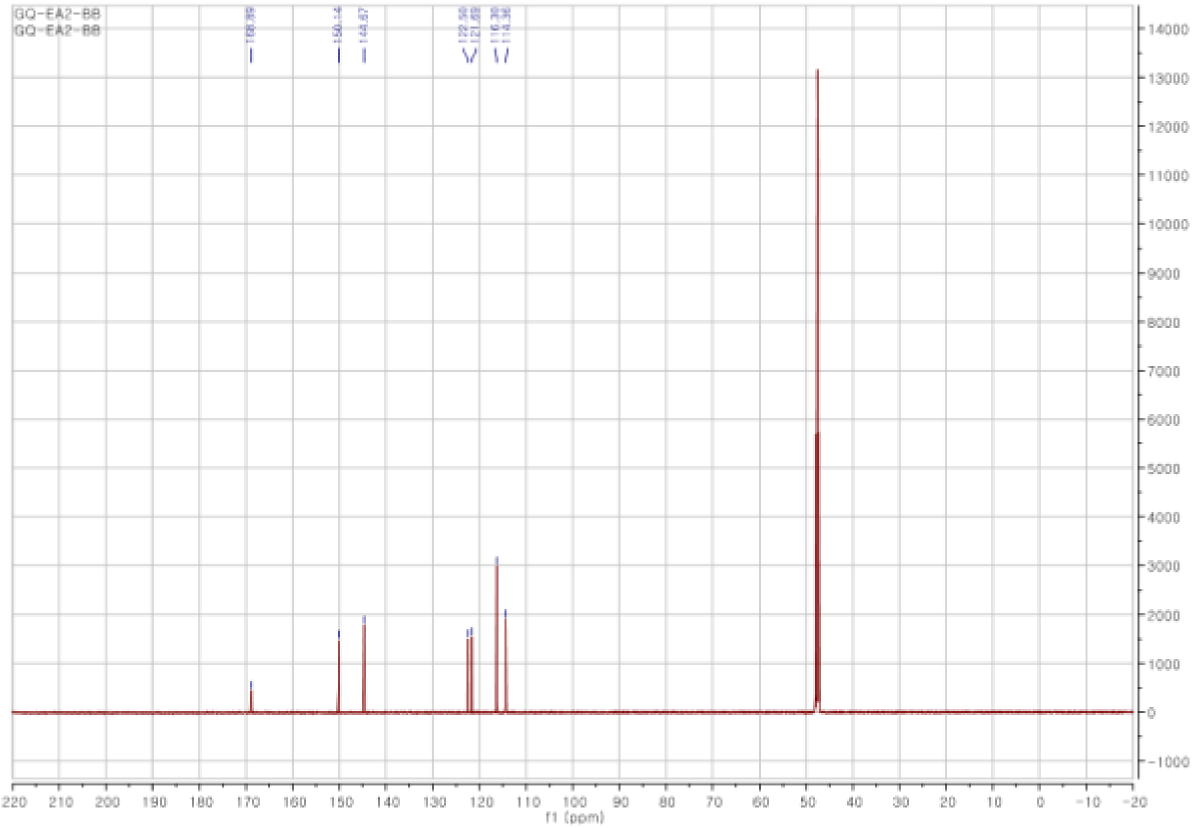

S3. ^{1}H and ^{13}C NMR spectrum of compound **2** in methanol-d_4 (600 and 150 MHz)

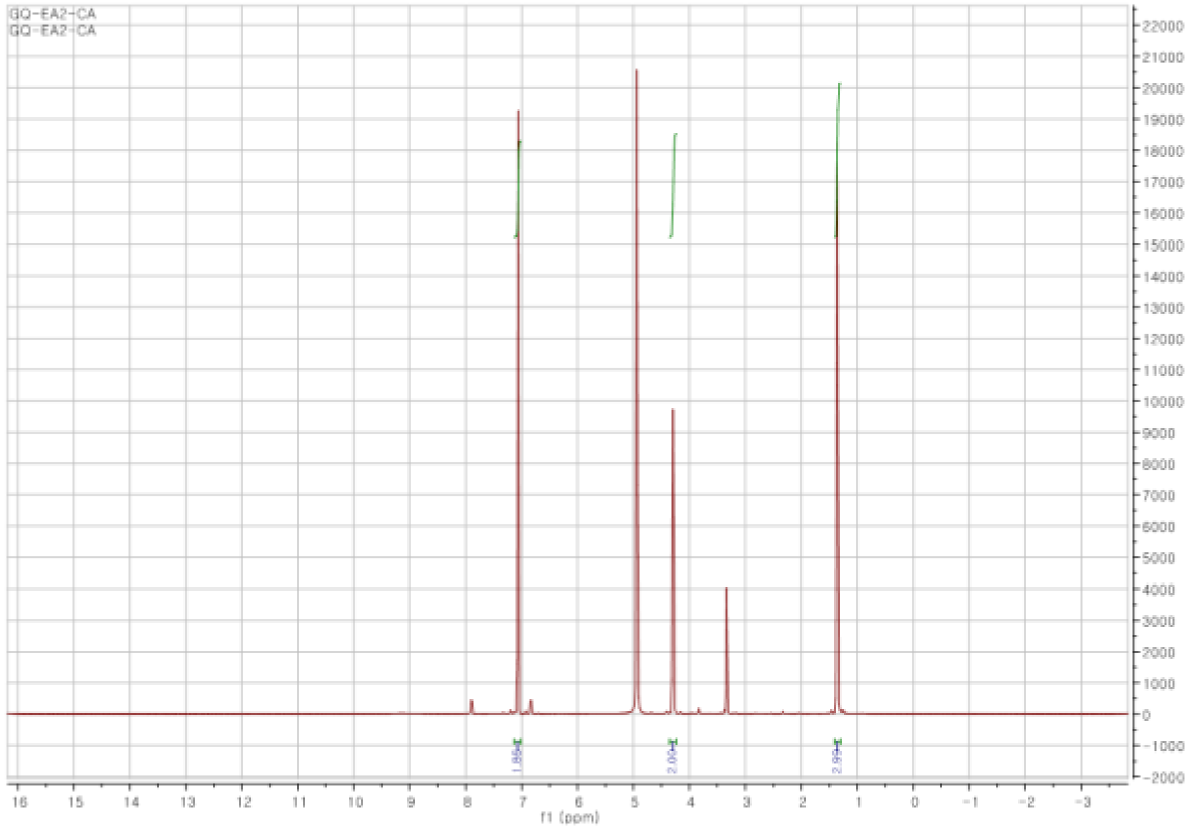

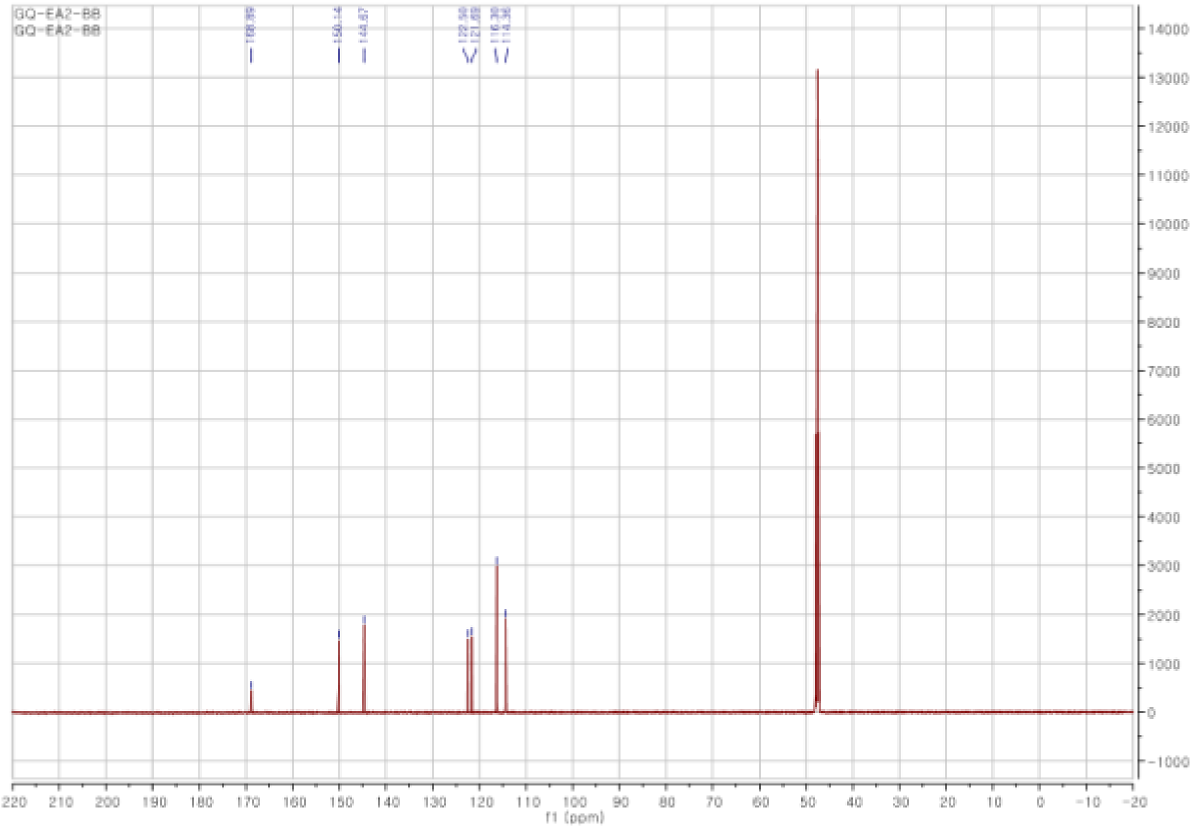

S4. ^{1}H and ^{13}C NMR spectrum of compound **3** in methanol-d_4 (600 and 150 MHz)

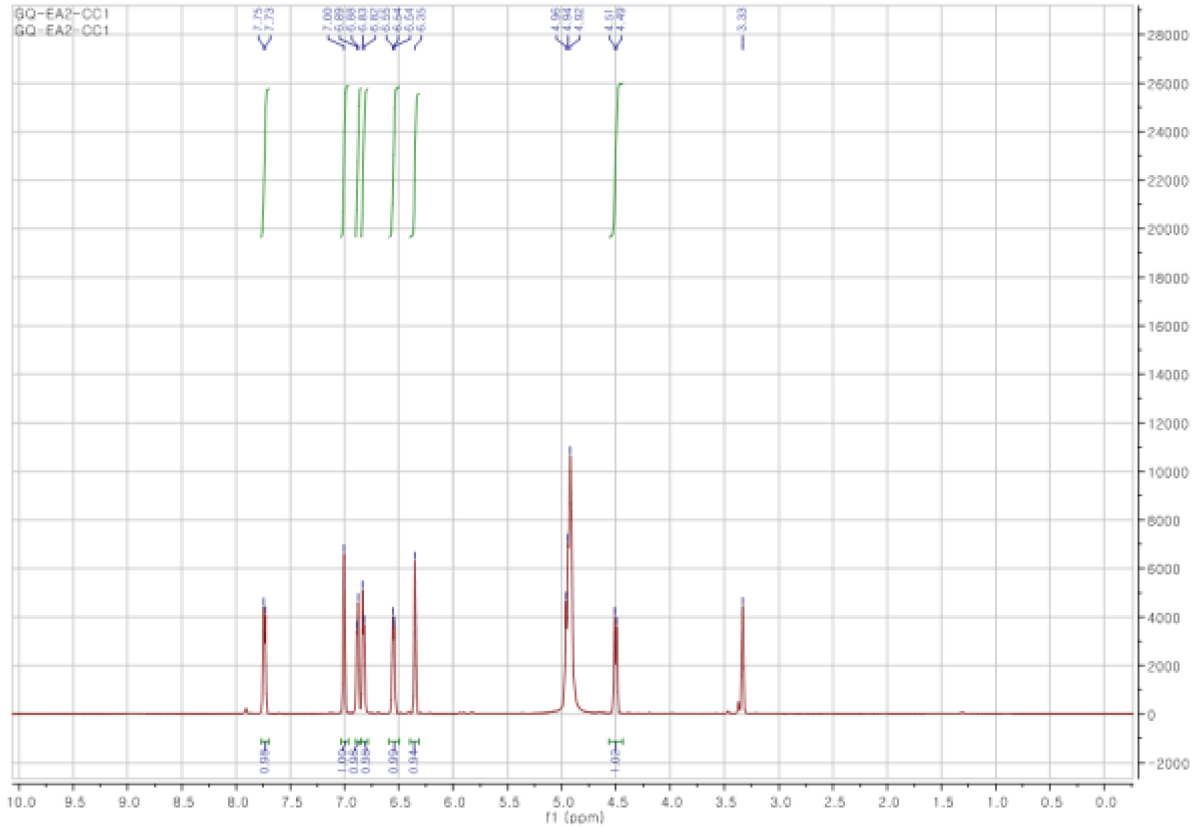

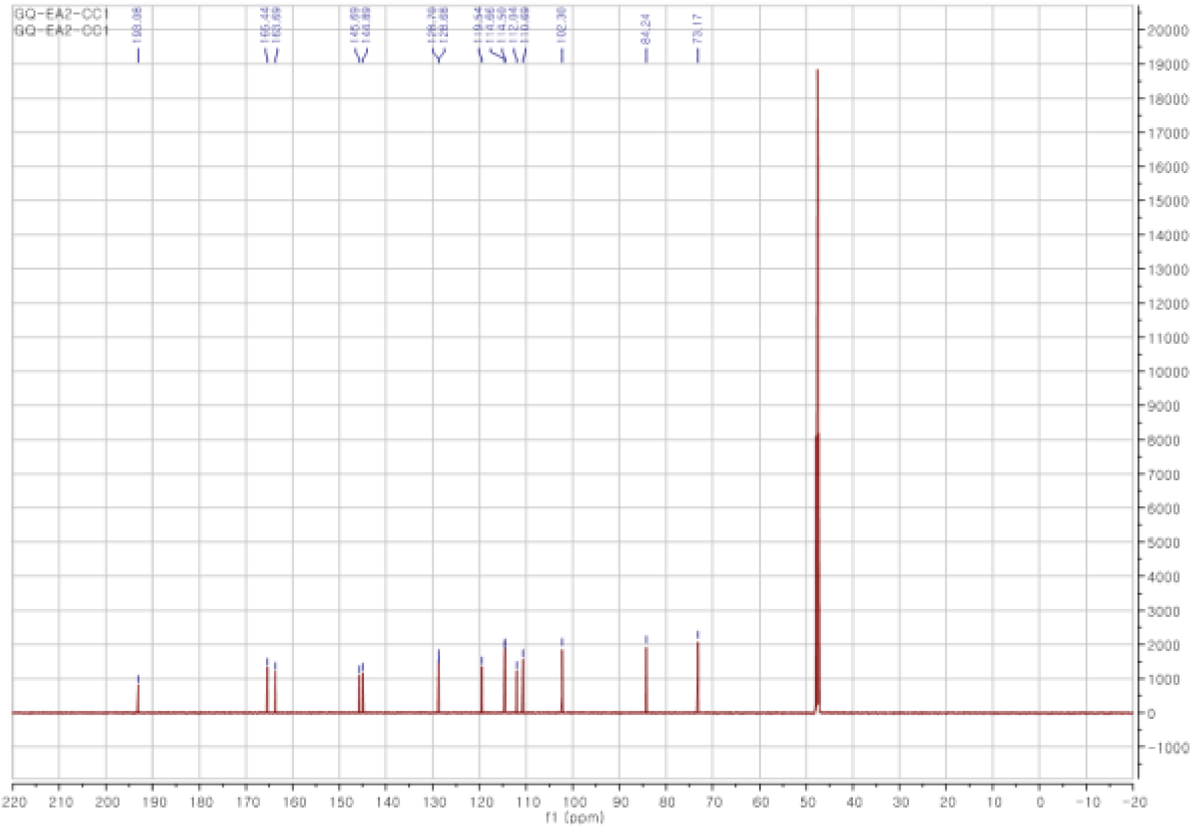

S5. ^{1}H and ^{13}C NMR spectrum of compound **4** in methanol-d_4 (600 and 150 MHz)

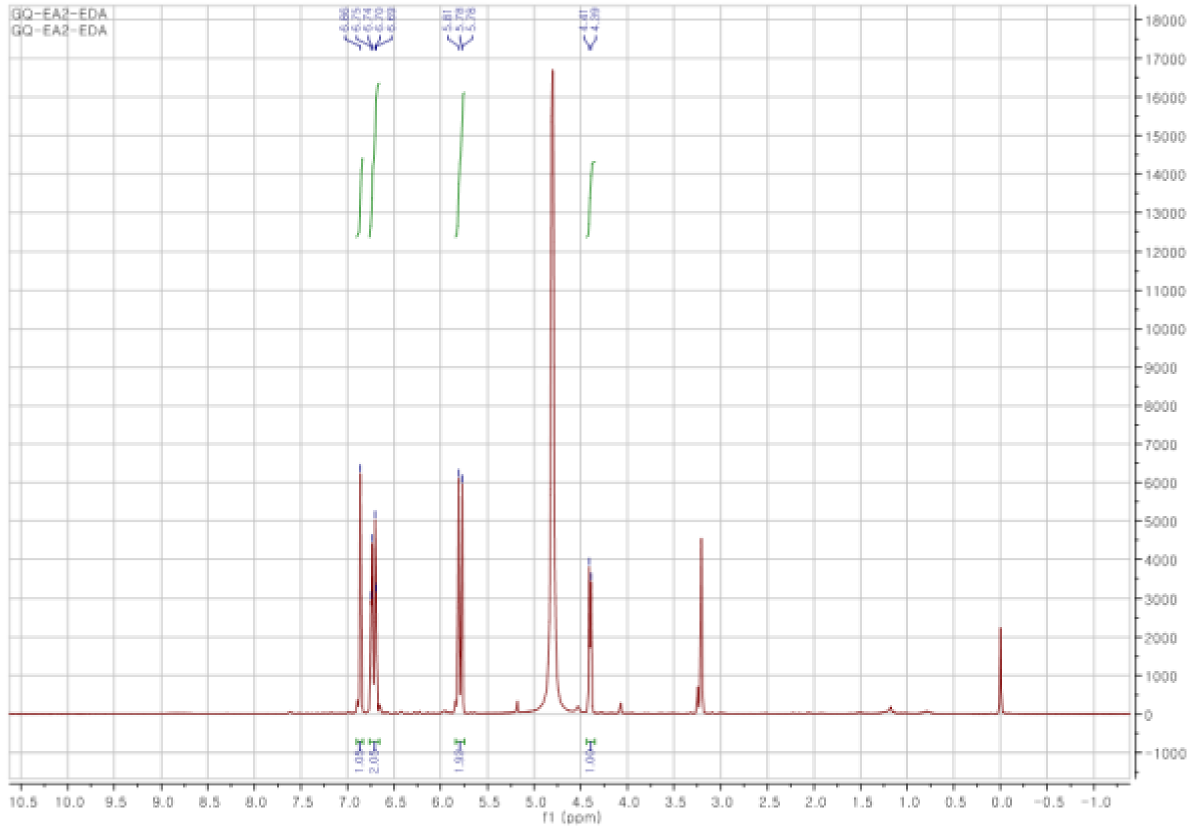

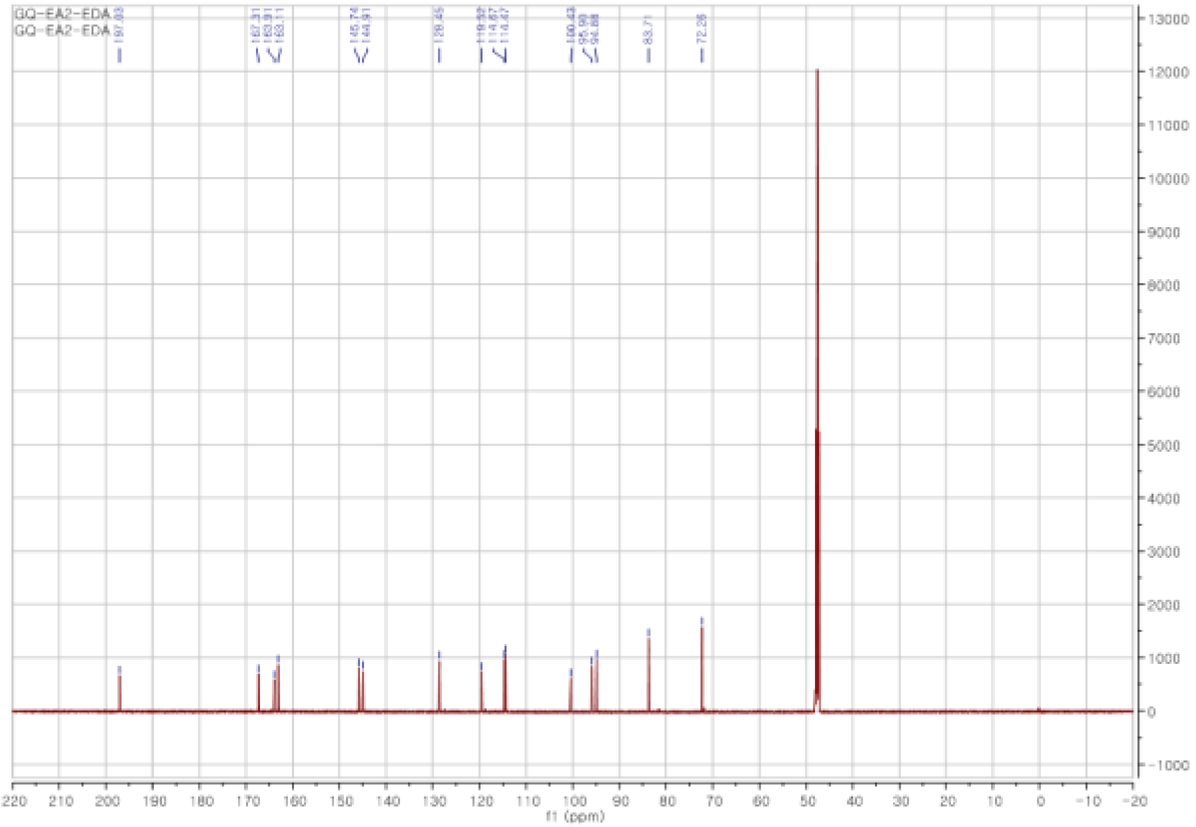

S5. ^{1}H and ^{13}C NMR spectrum of compound **4** in methanol-d_4 (600 and 150 MHz)

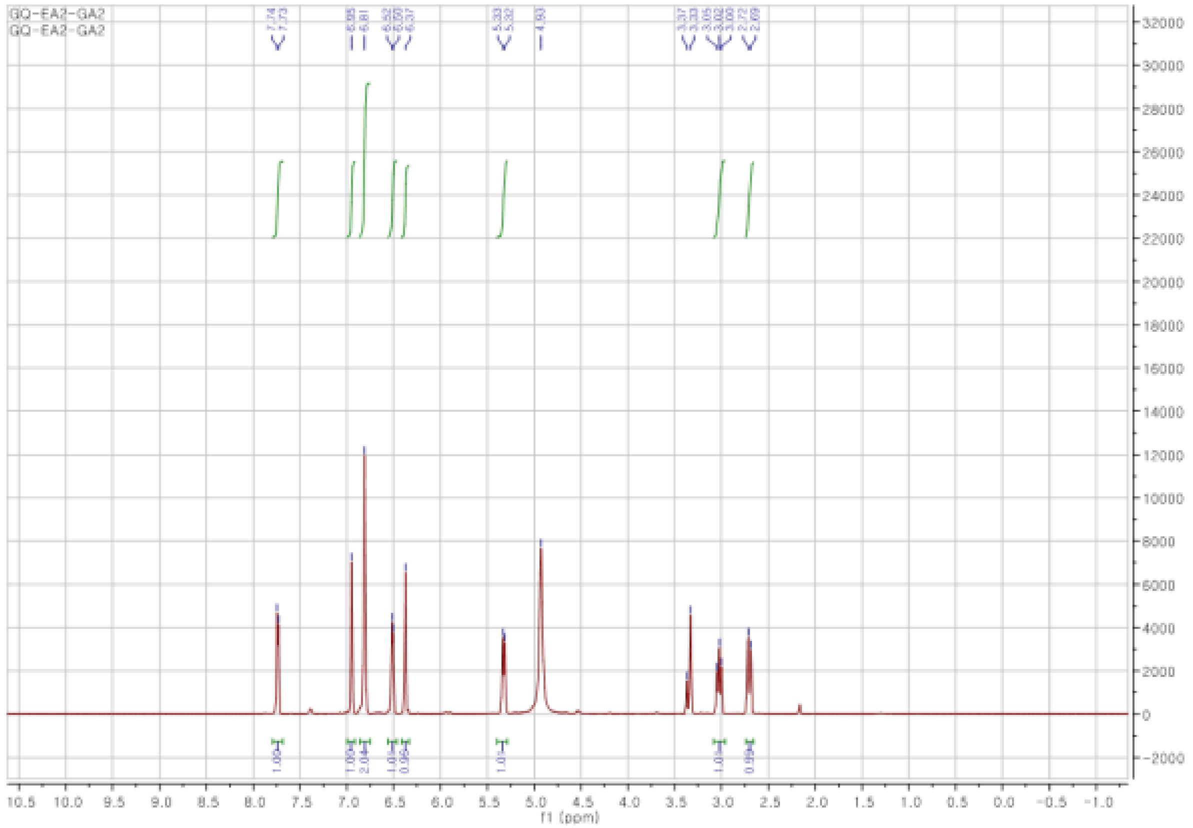

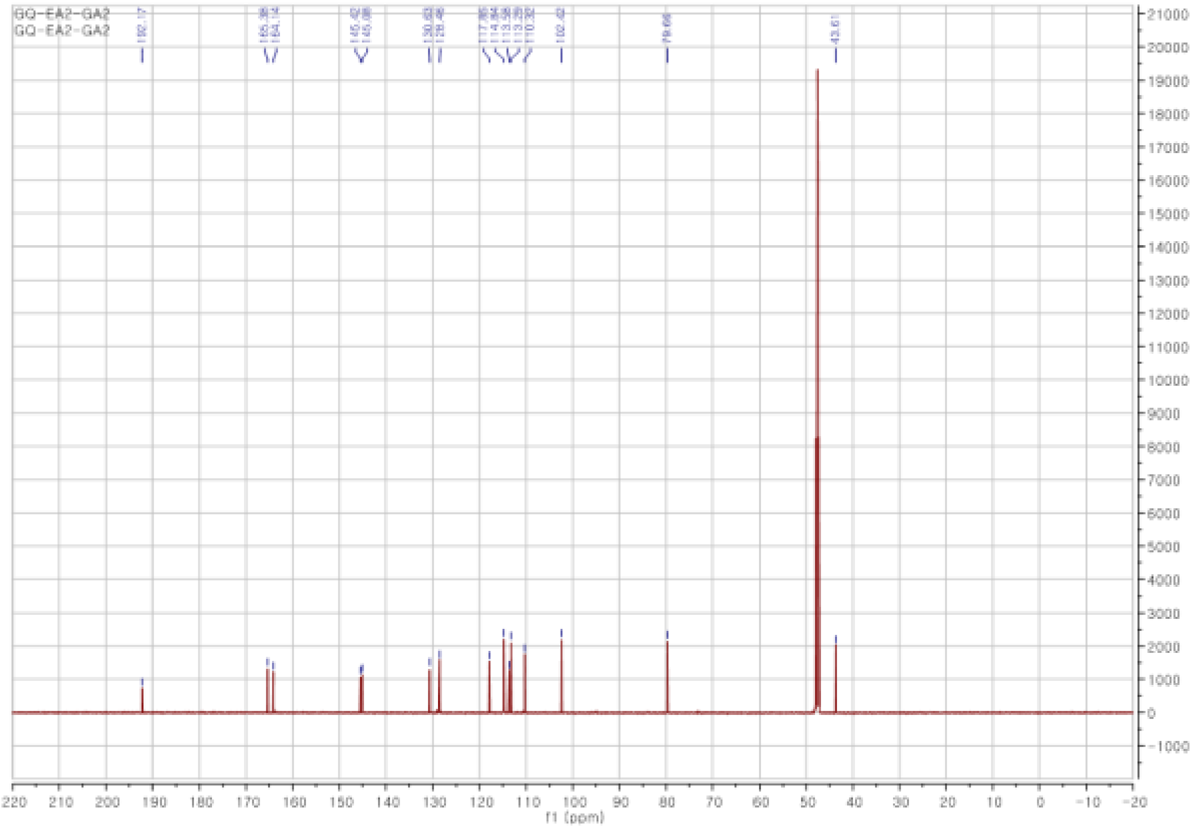

S6. ^{1}H and ^{13}C NMR spectrum of compound **5** in methanol-d_4 (600 and 150 MHz)

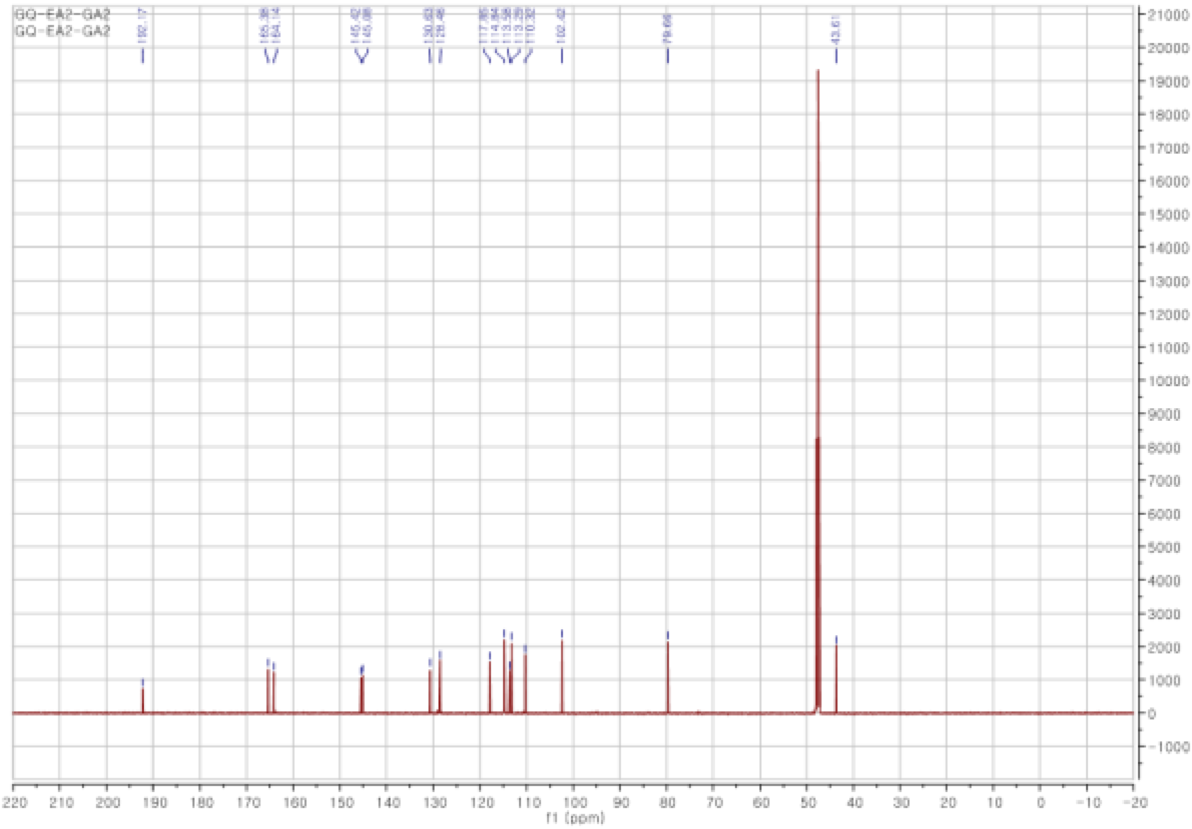

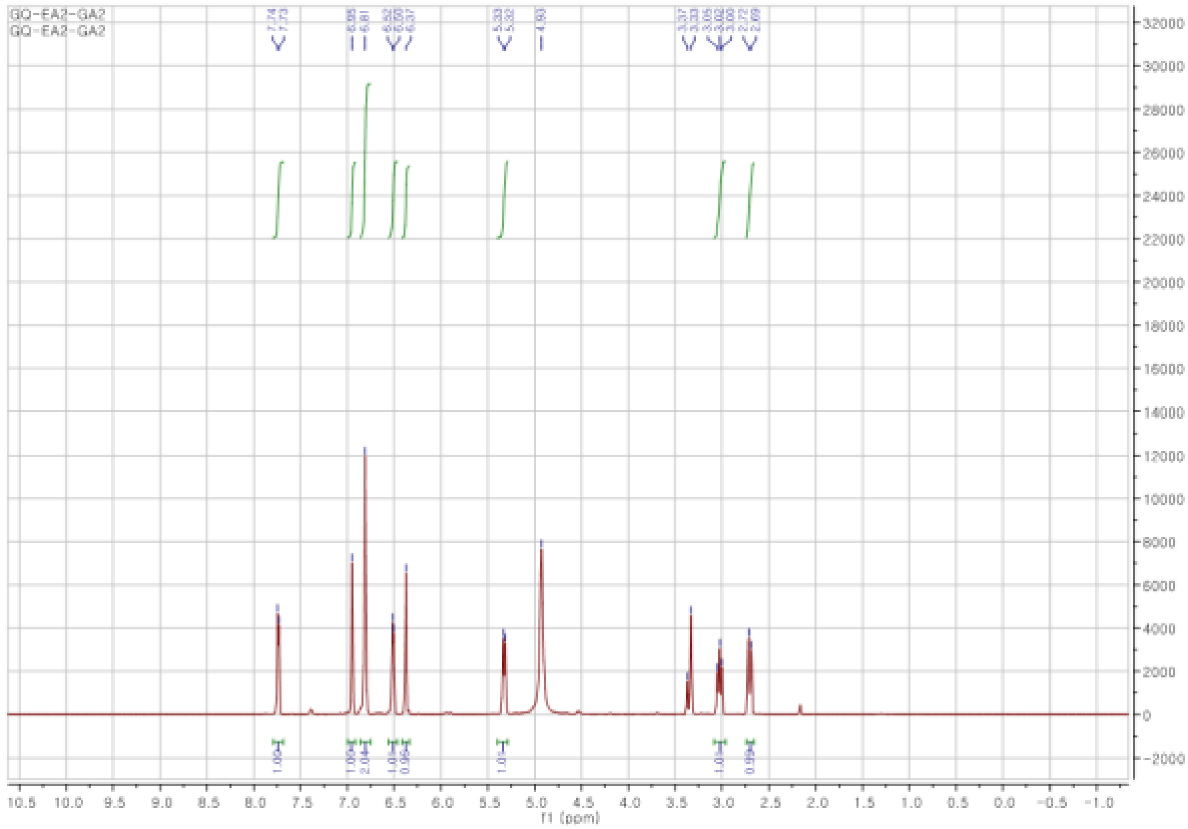

S7. ^{1}H and ^{13}C NMR spectrum of compound **6** in methanol-d_4 (600 and 150 MHz)

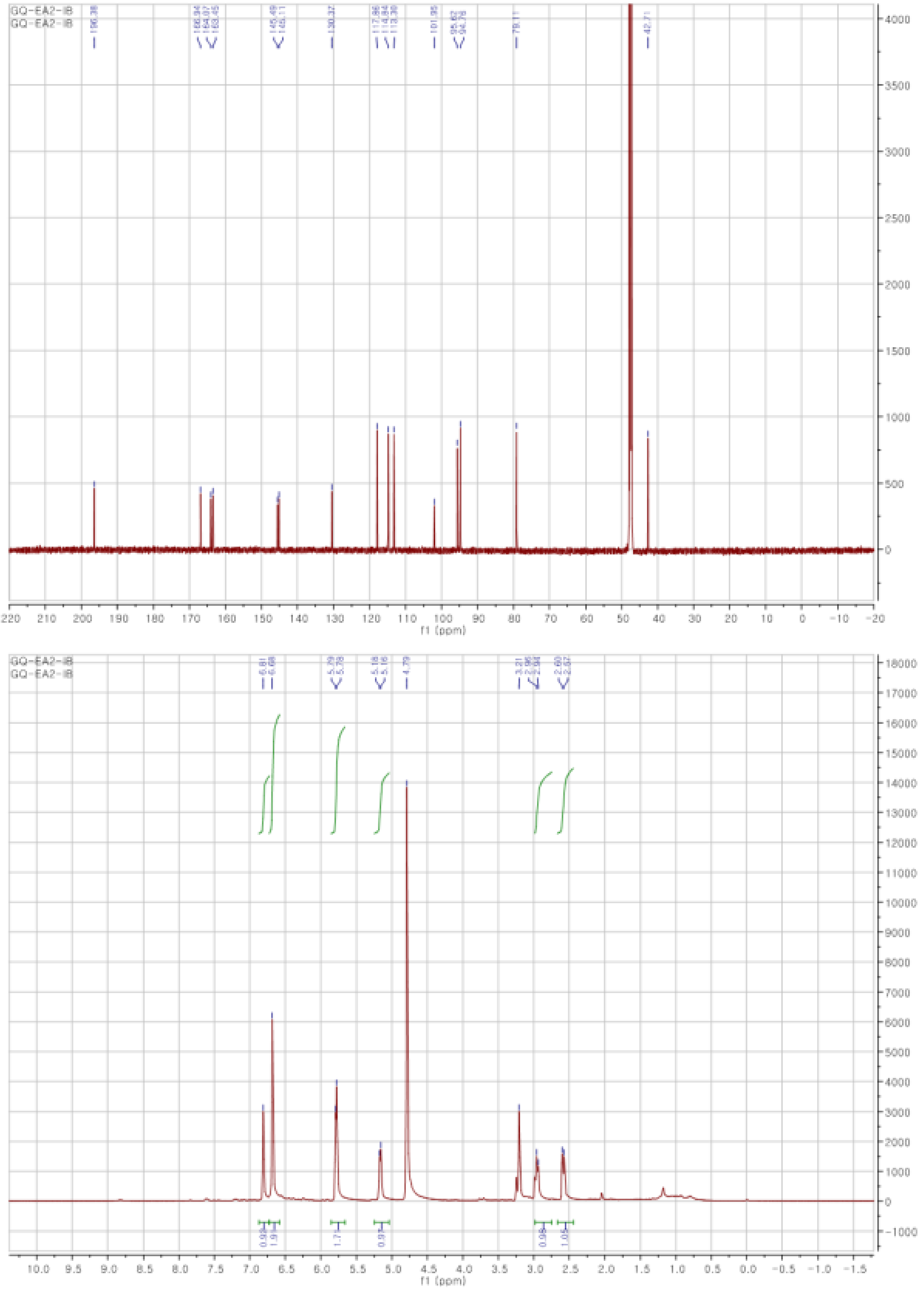

S8. ^{1}H and ^{13}C NMR spectrum of compound **7** in methanol-d_4 (600 and 150 MHz)

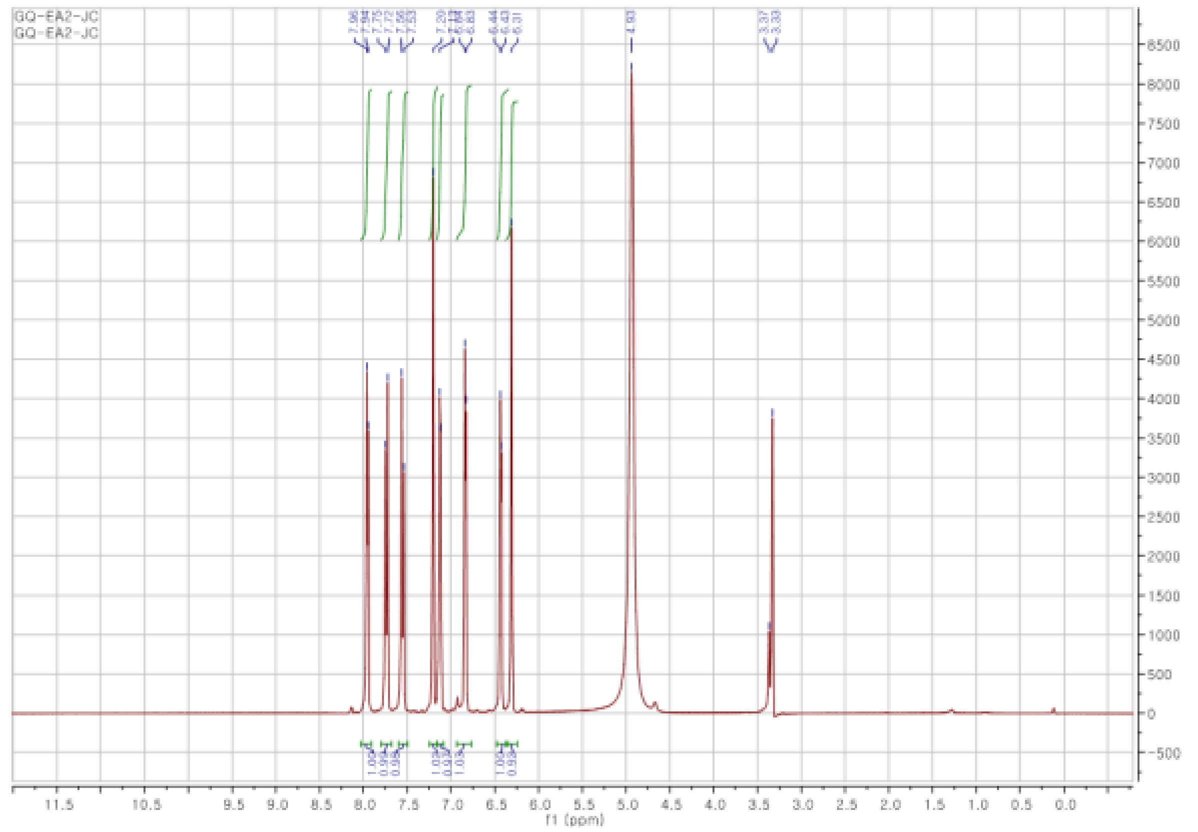

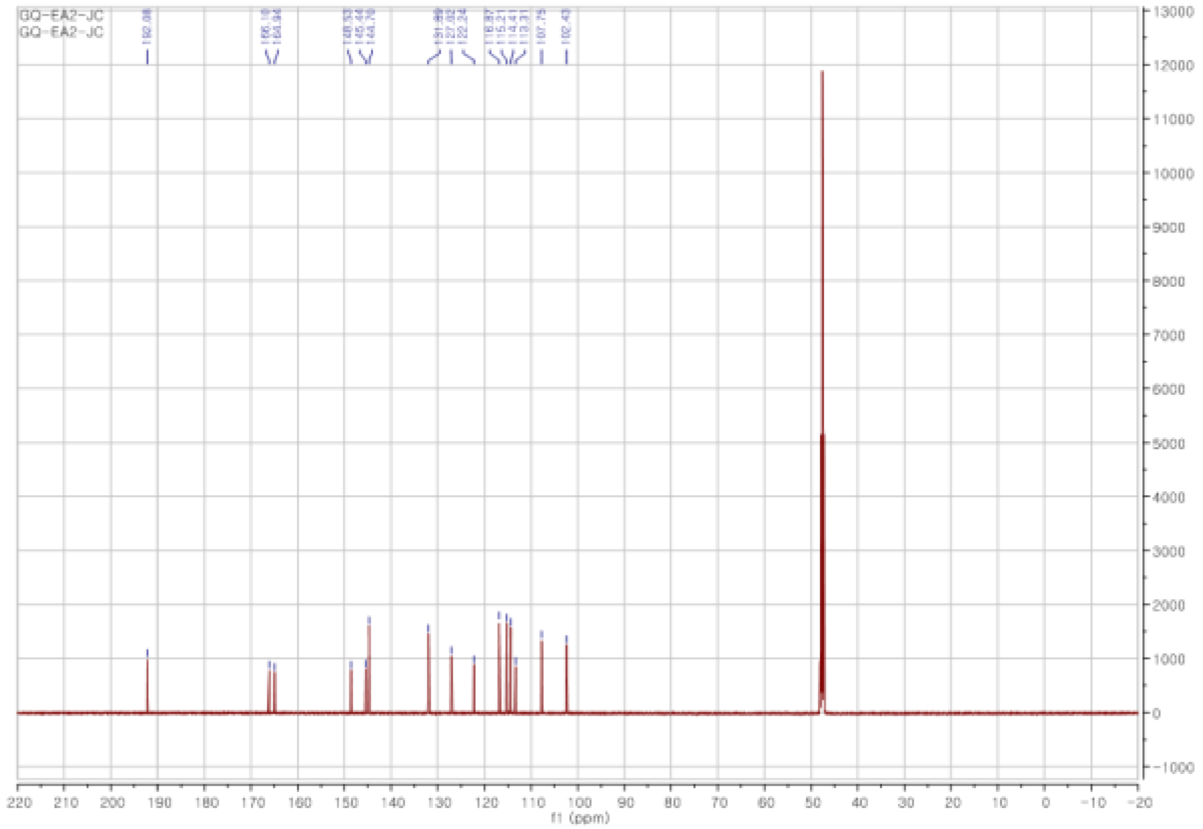

S9. ^{1}H and ^{13}C NMR spectrum of compound **8** in methanol-d_4 (600 and 150 MHz)

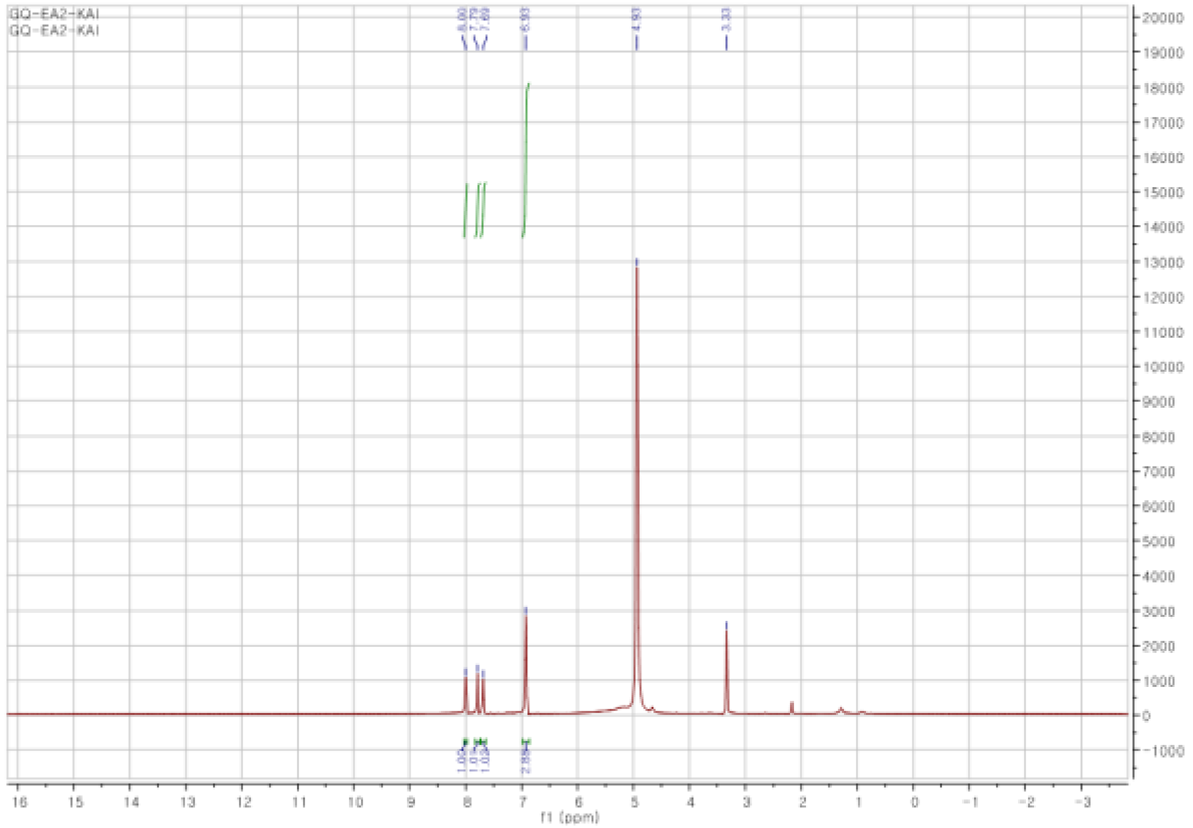

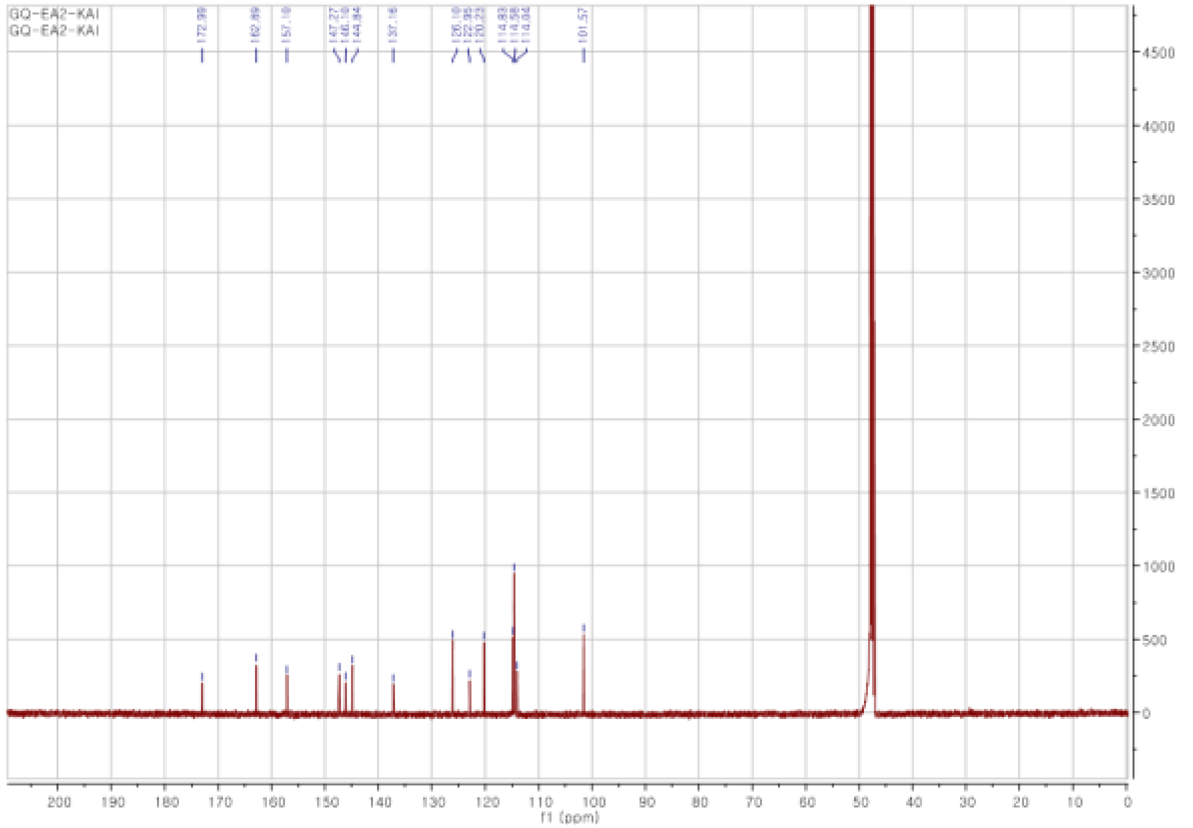

S10. ^{1}H and ^{13}C NMR spectrum of compound **9** in methanol-d_4 (600 and 150 MHz)

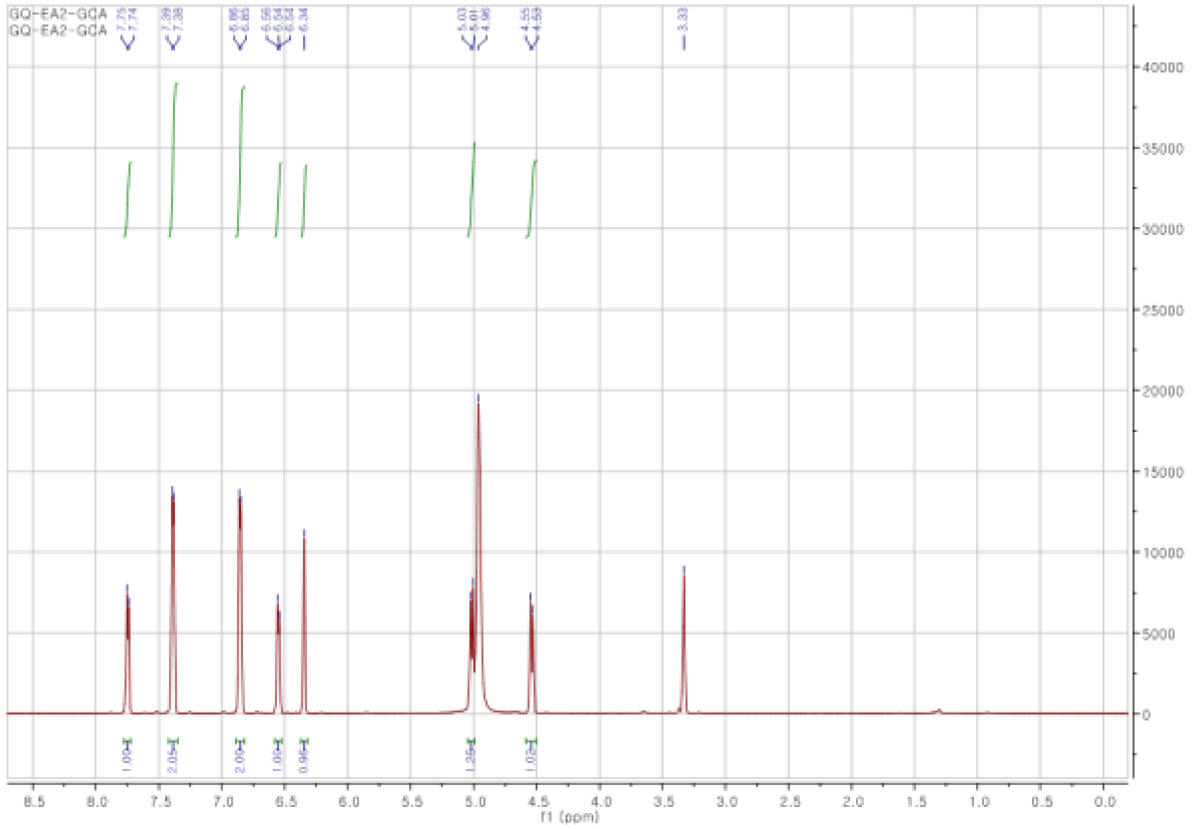

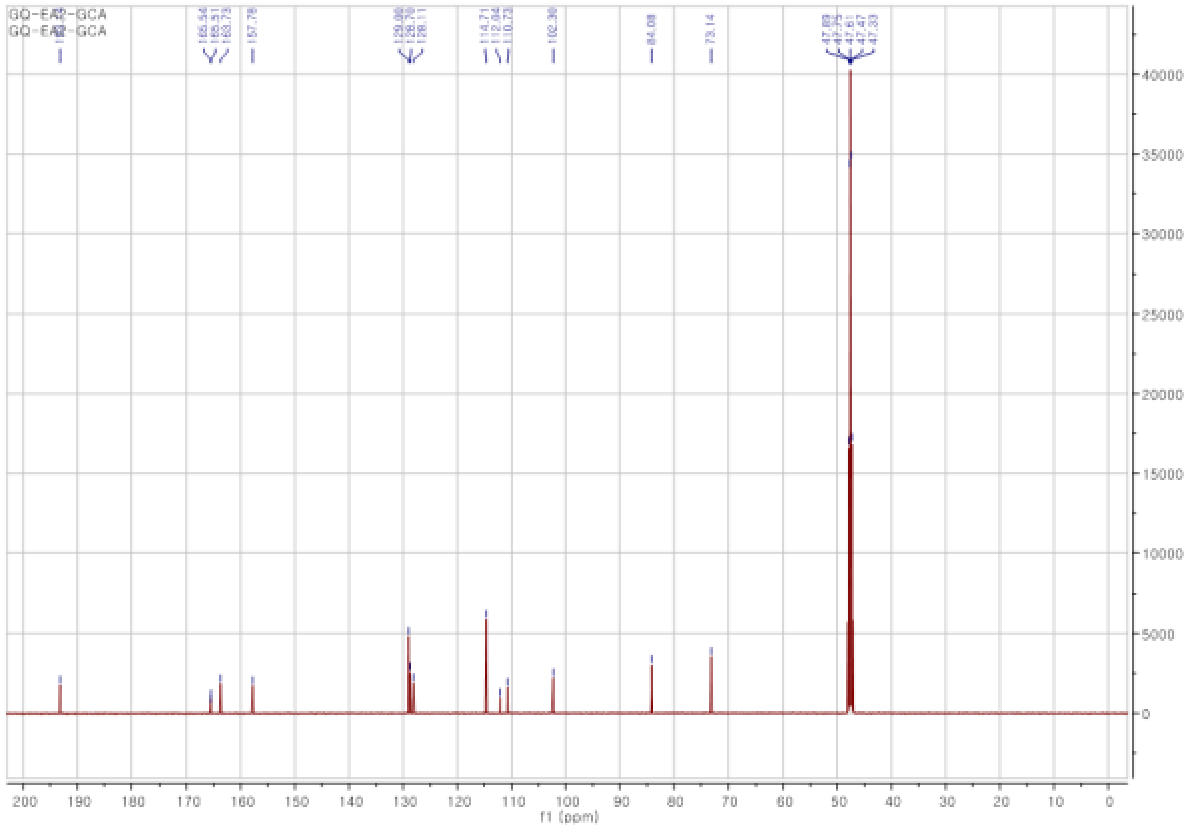

S11. ^{1}H and ^{13}C NMR spectrum of compound **10** in methanol-d_4 (600 and 150 MHz)

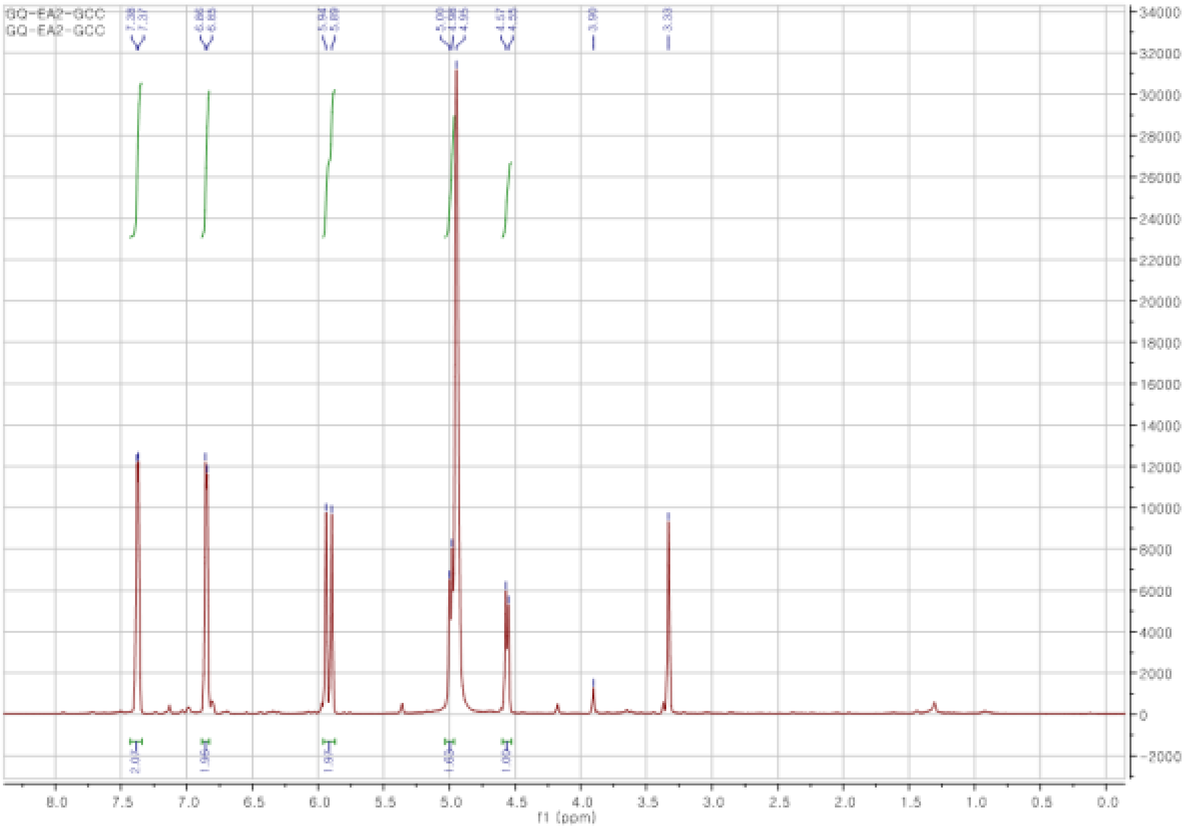

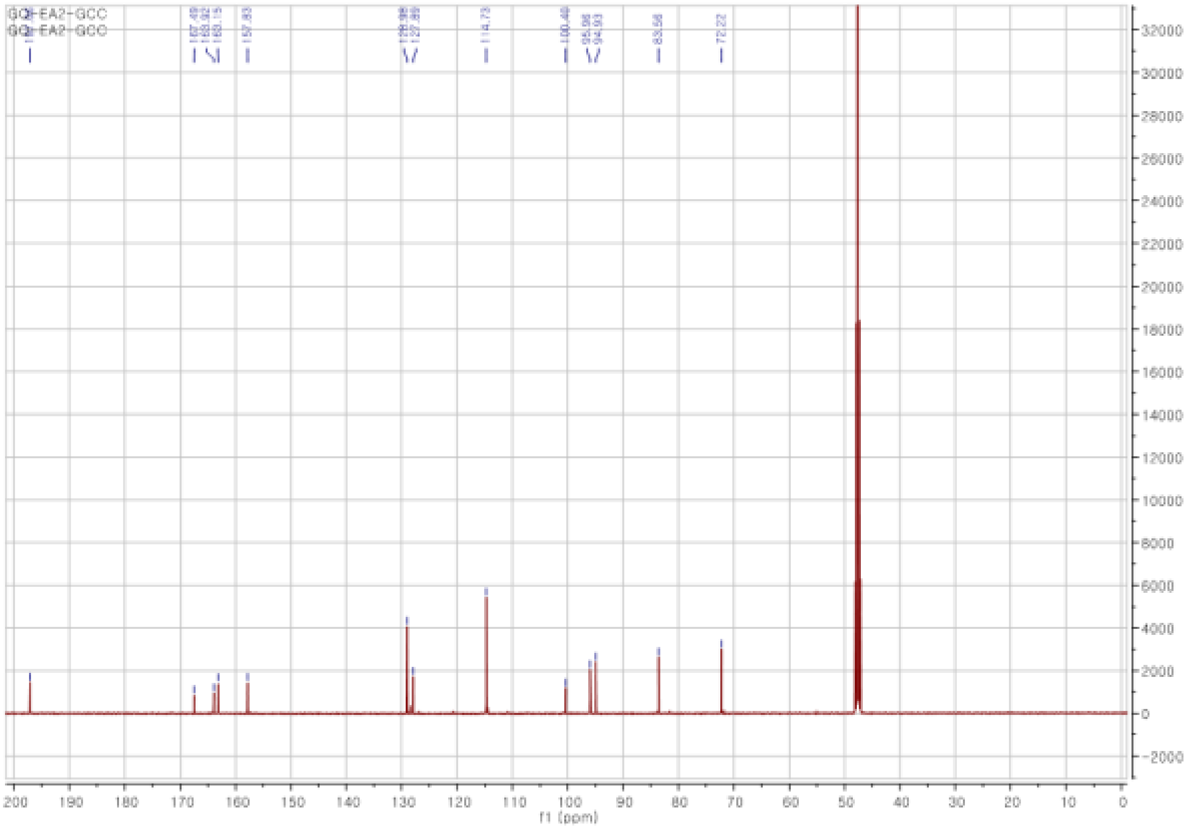

S12. ^{1}H and ^{13}C NMR spectrum of compound **11** in methanol-d_4 (600 and 150 MHz)

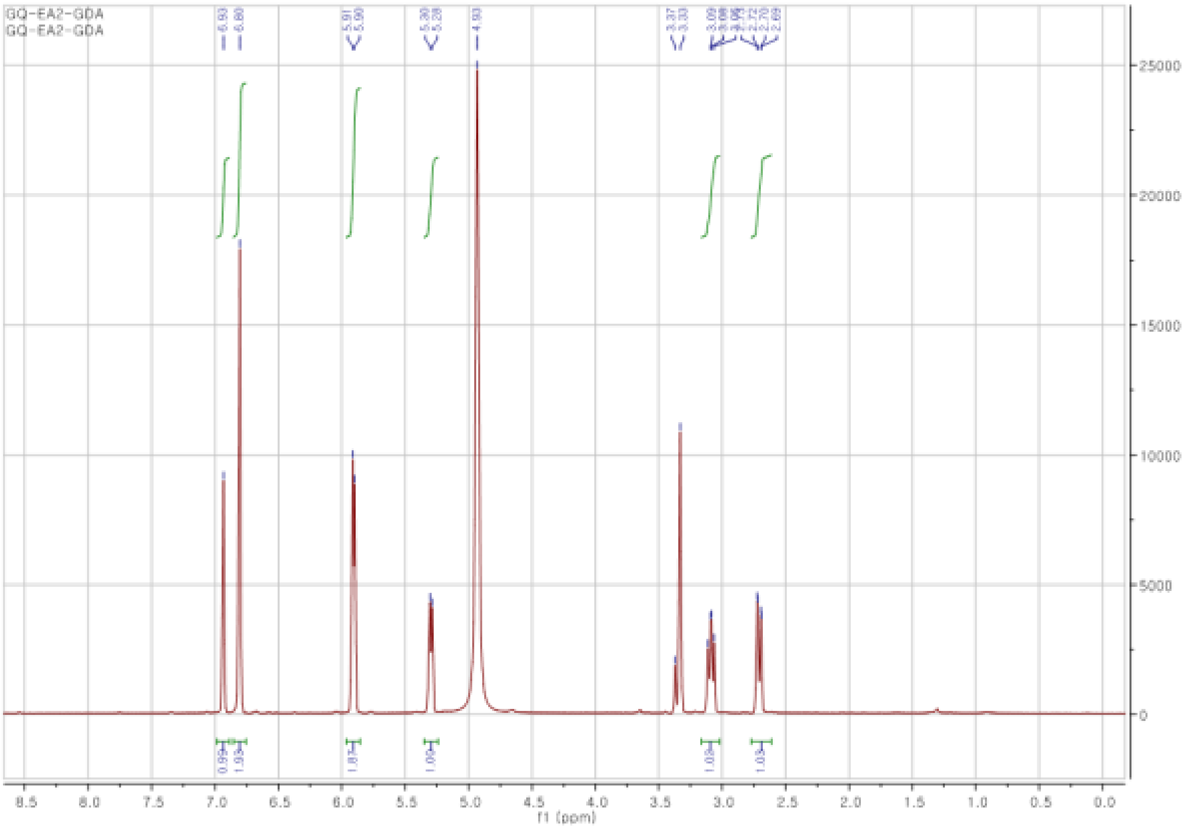

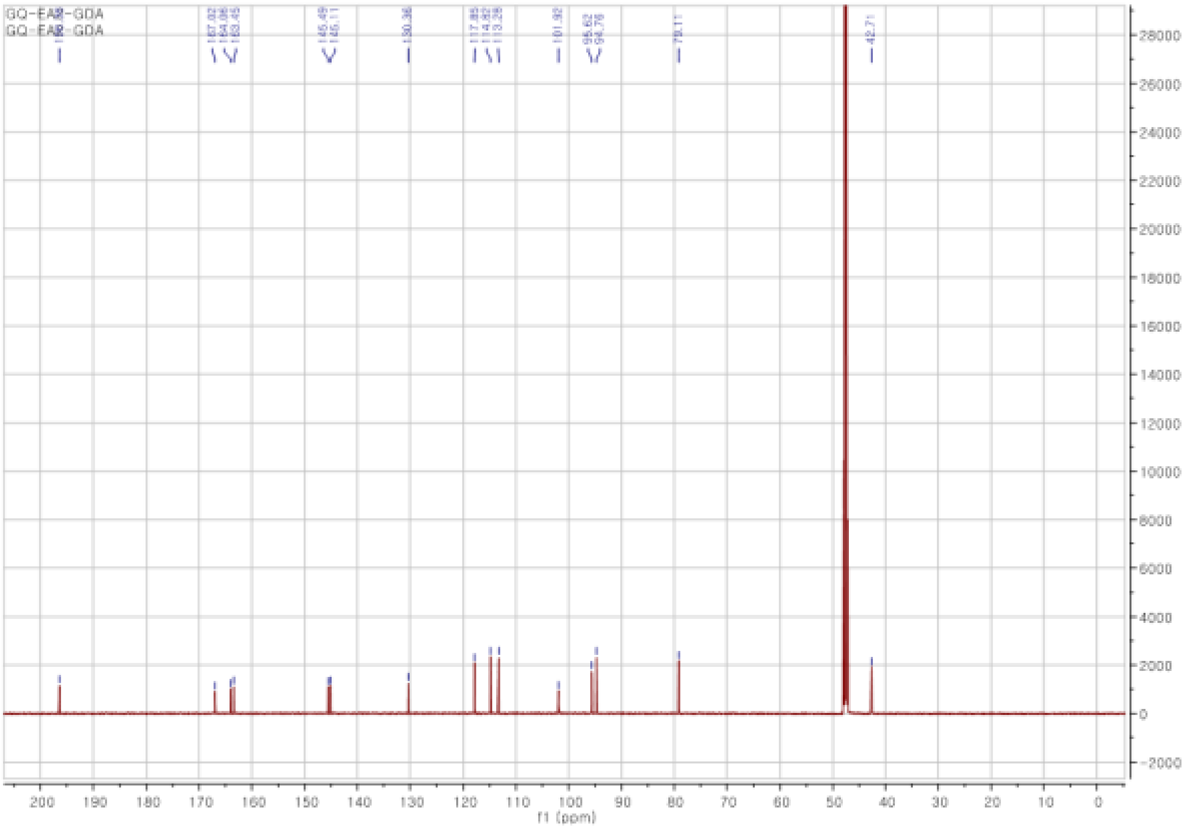

S13. ^{1}H and ^{13}C NMR spectrum of compound **12** in methanol-d_4 (600 and 150 MHz)

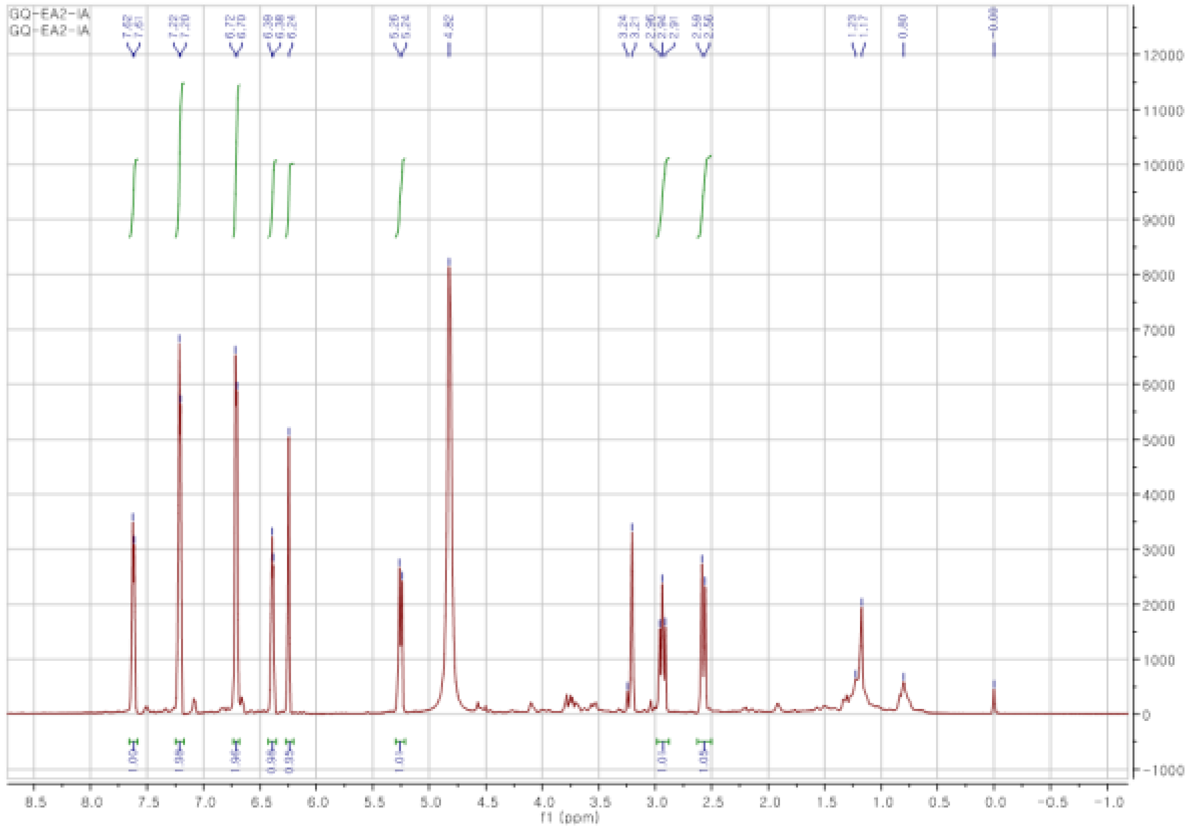

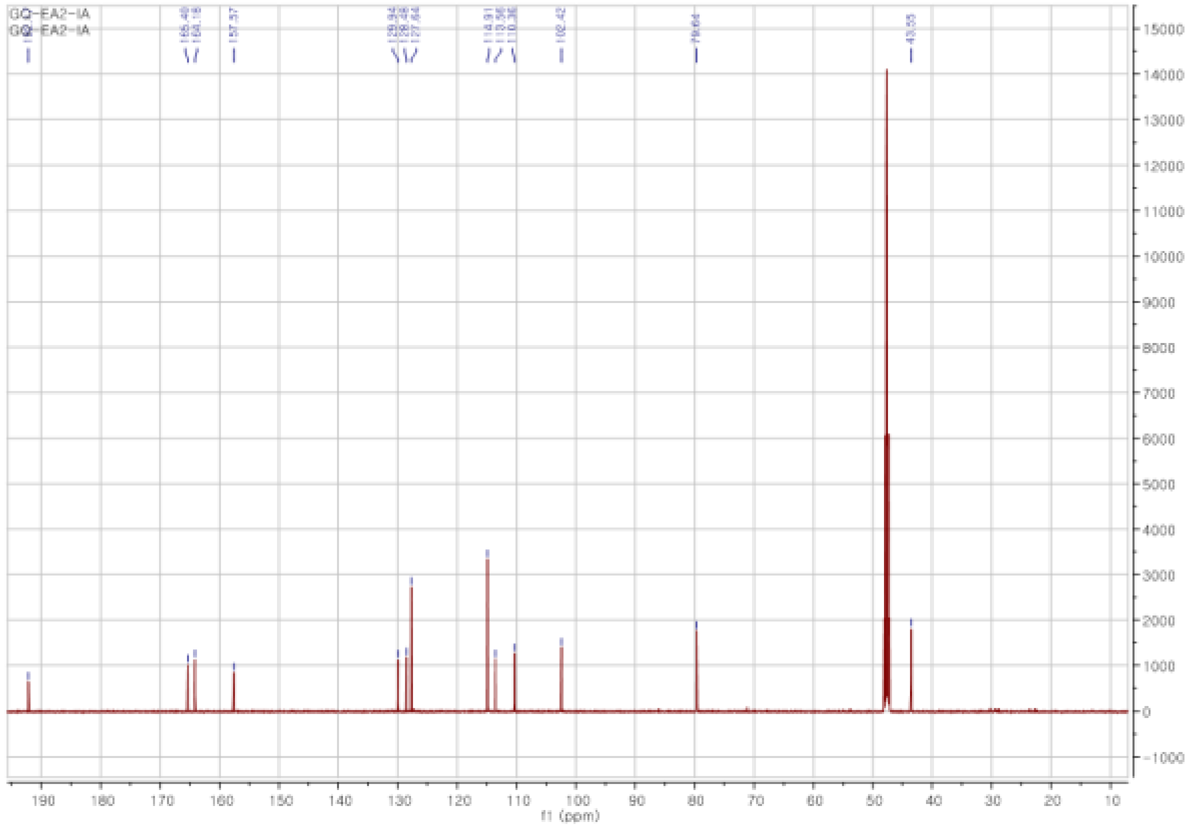

S14. ^{1}H and ^{13}C NMR spectrum of compound **13** in methanol-d_4 (600 and 150 MHz)

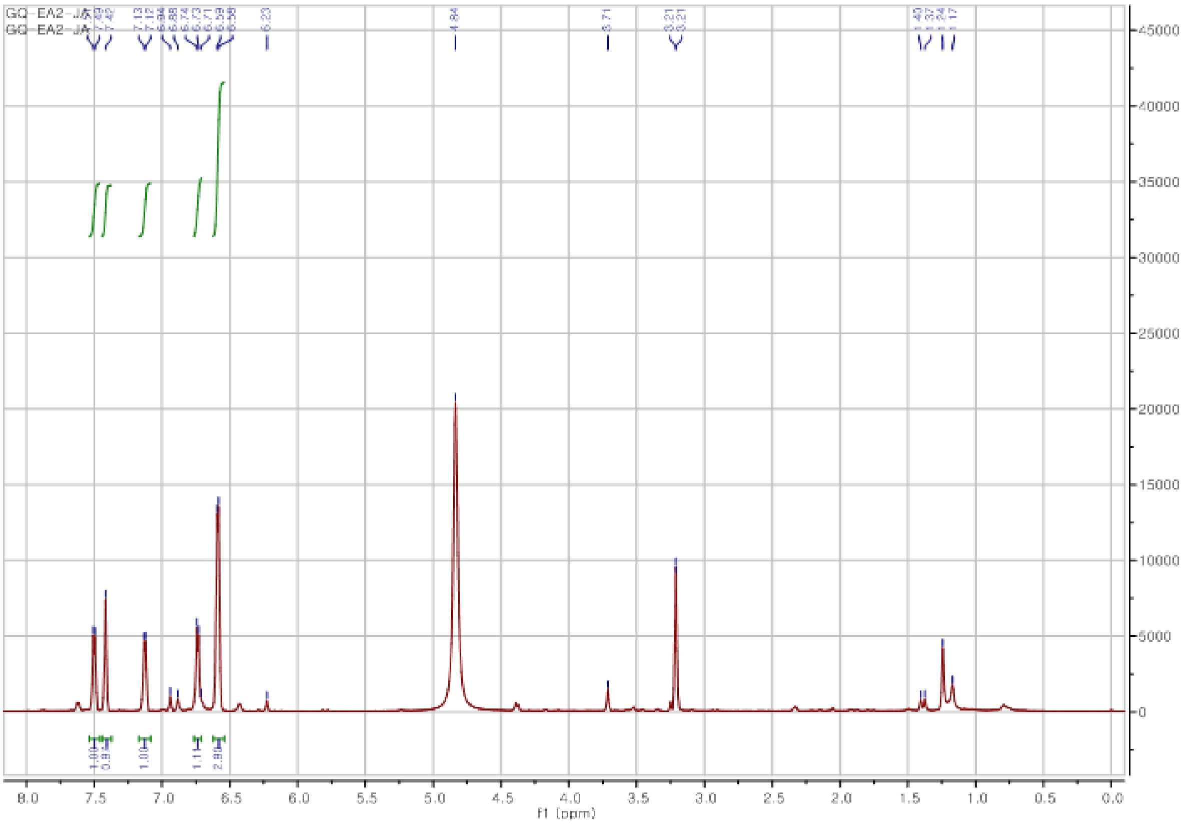

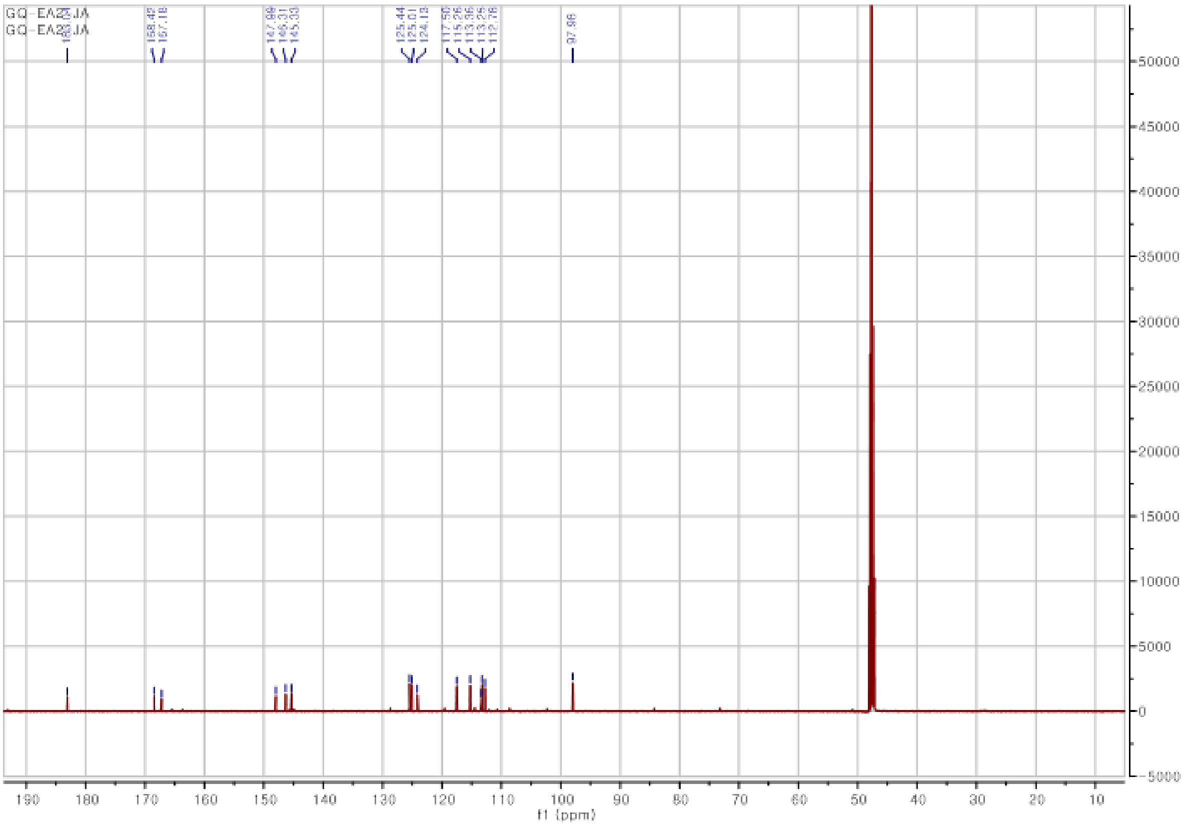

S15. ^{1}H and ^{13}C NMR spectrum of compound **14** in methanol-d_4 (600 and 150 MHz)

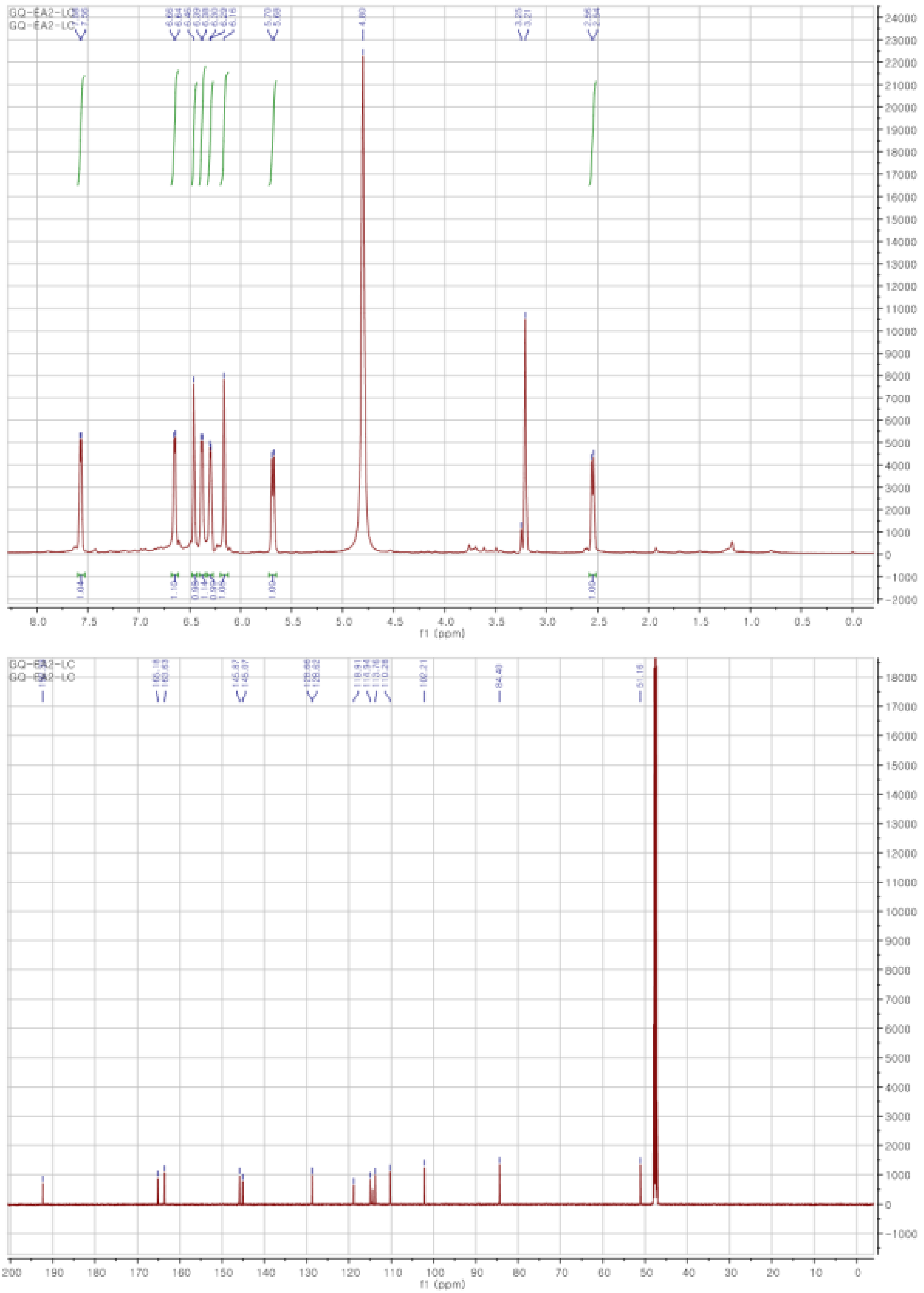

S16. ^{1}H and ^{13}C NMR spectrum of compound **15** in methanol-d_4 (600 and 150 MHz)

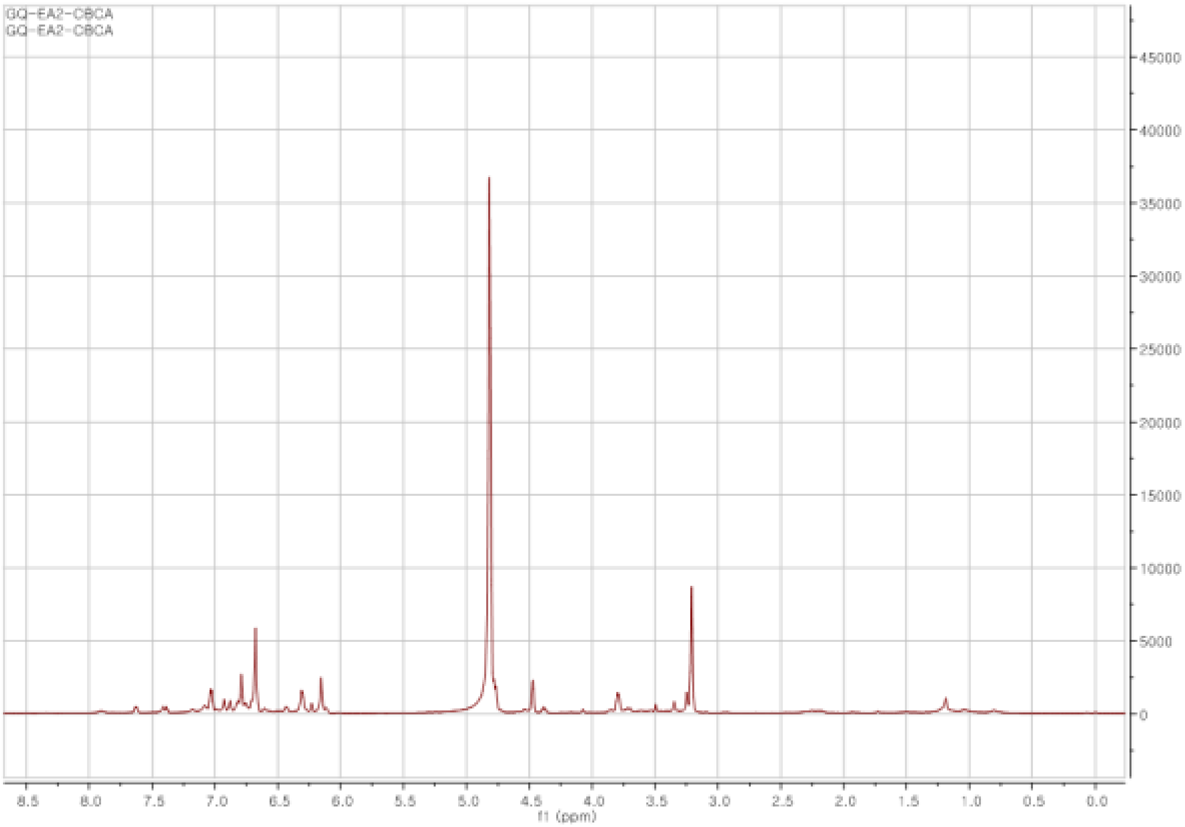

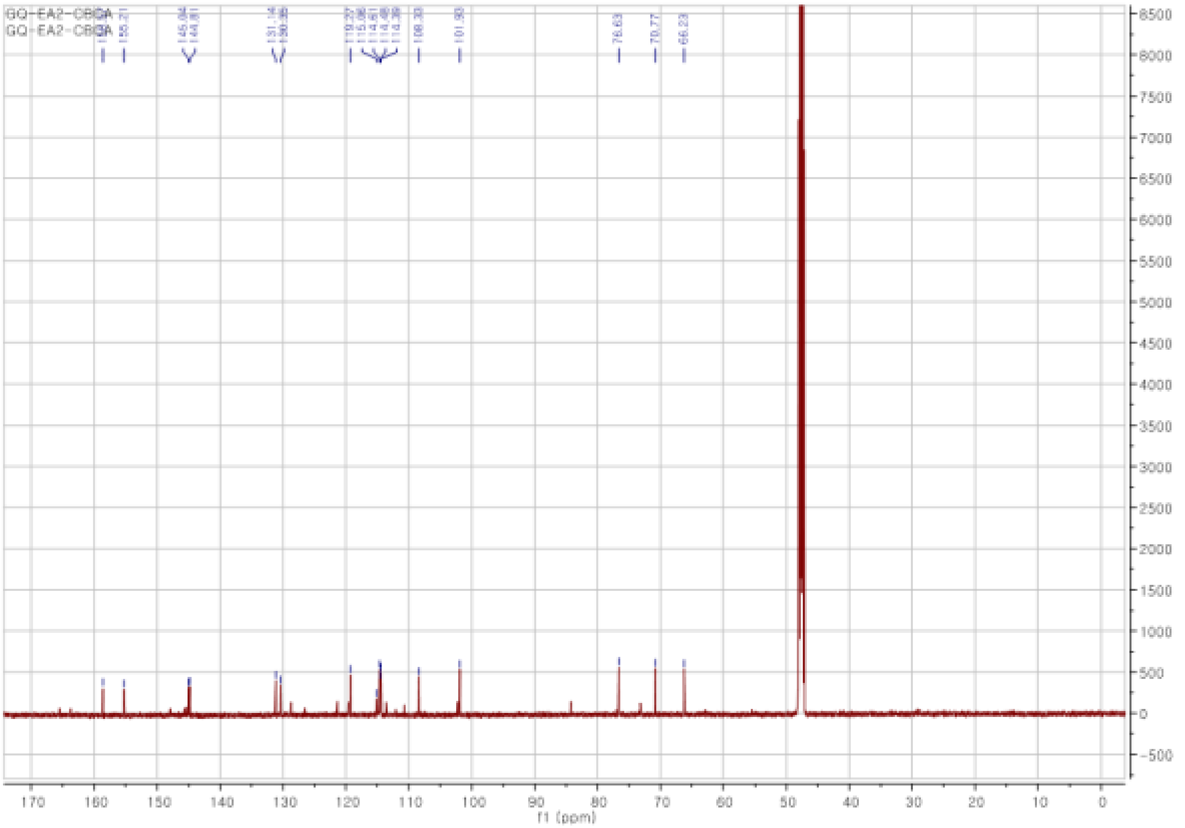

S17. ^{1}H and ^{13}C NMR spectrum of compound **16** in methanol-d_4 (600 and 150 MHz)

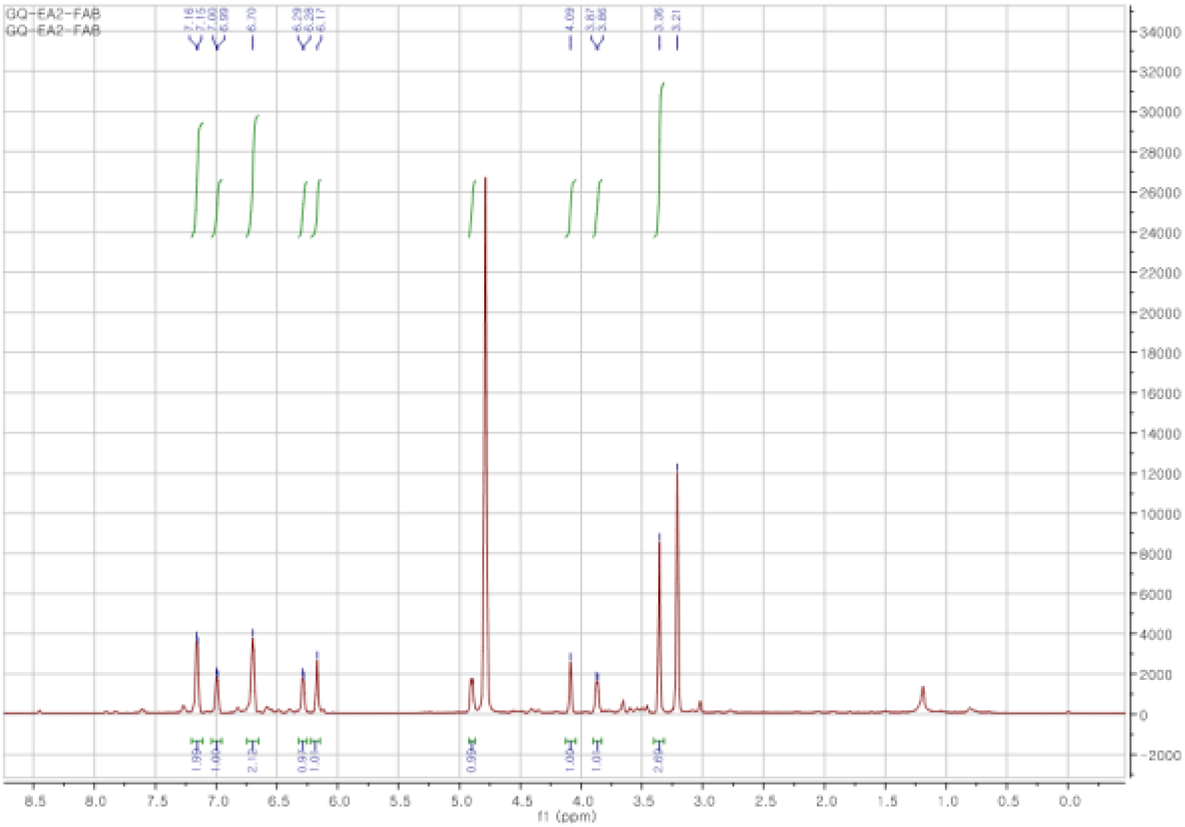

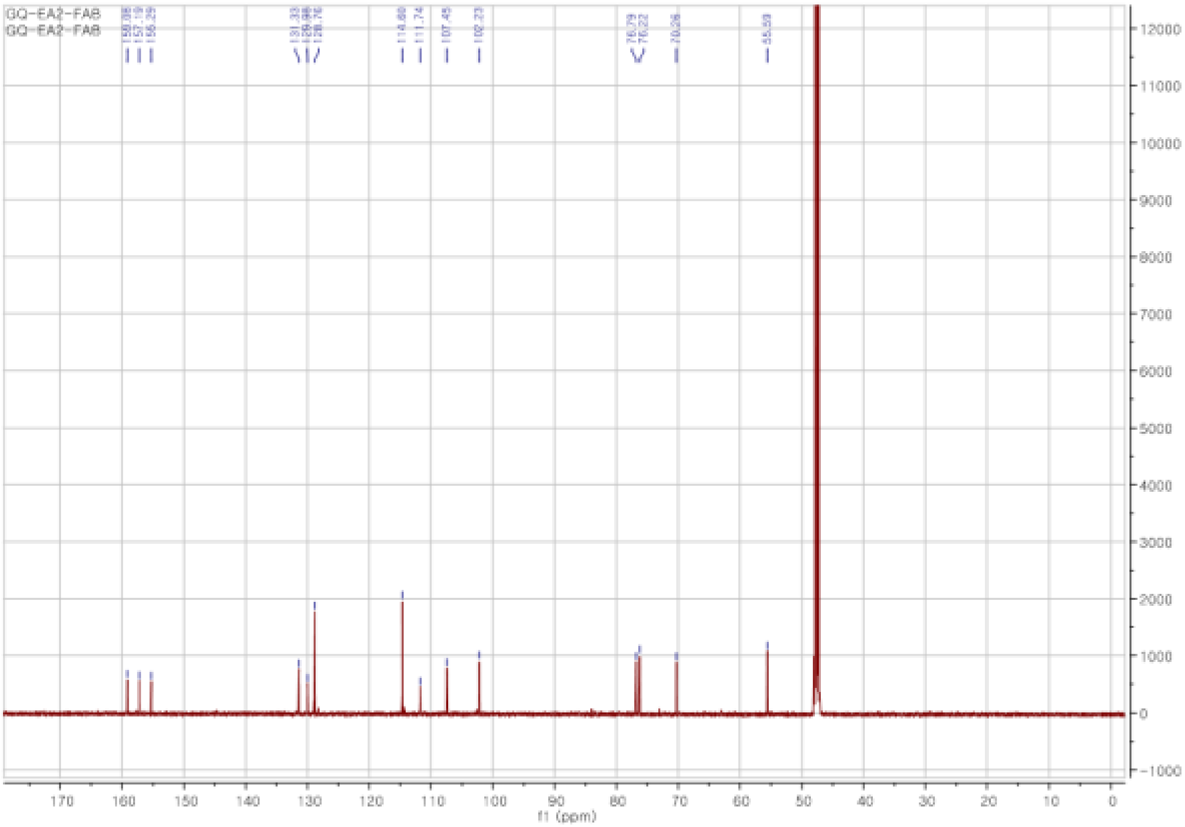

S18. ^{1}H and ^{13}C NMR spectrum of compound **17** in methanol-d_4 (600 and 150 MHz)

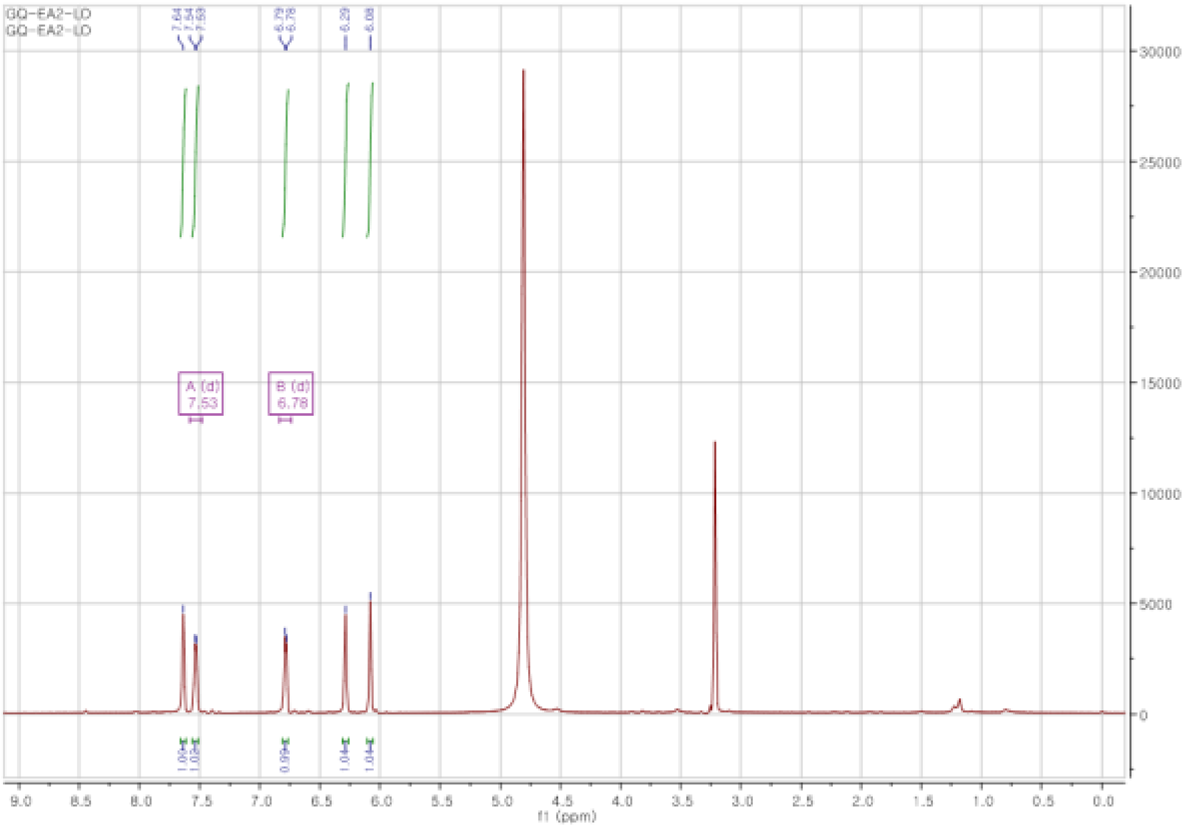

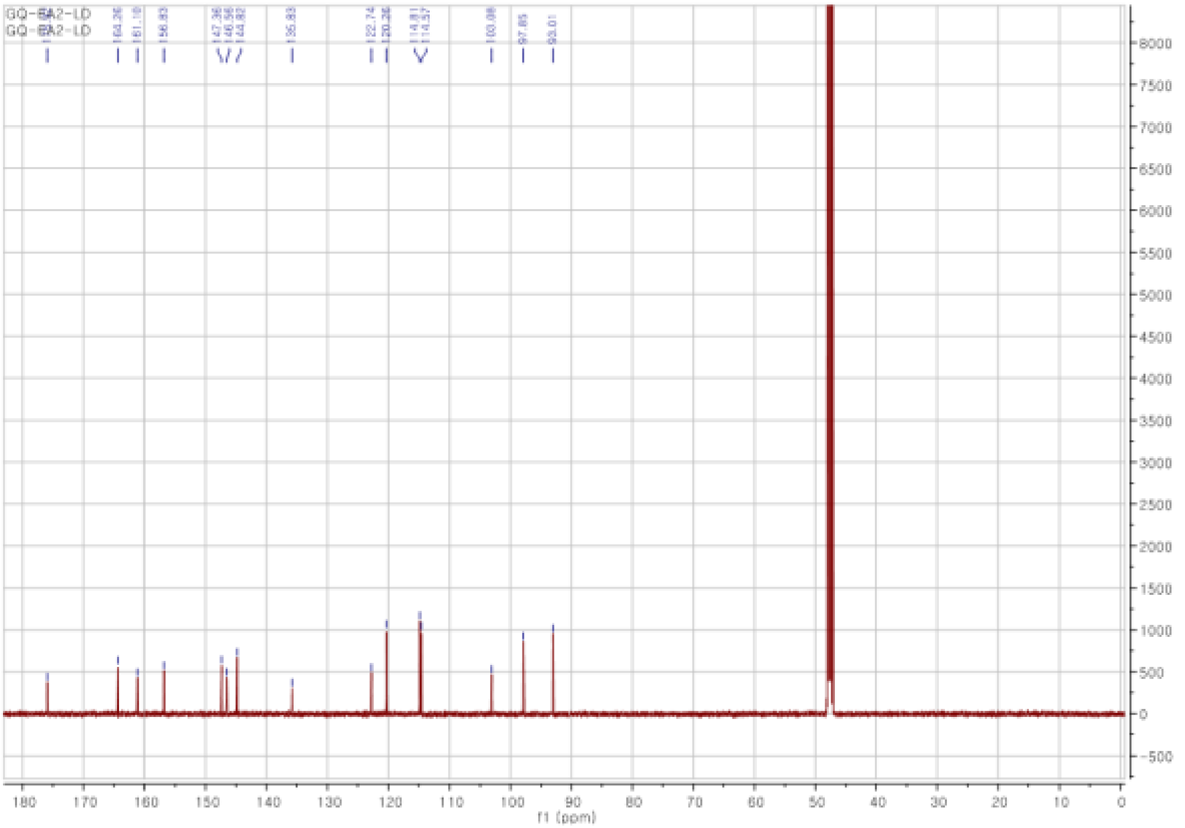

S19. ^{1}H and ^{13}C NMR spectrum of compound **18** in methanol-d_4 (600 and 150 MHz)

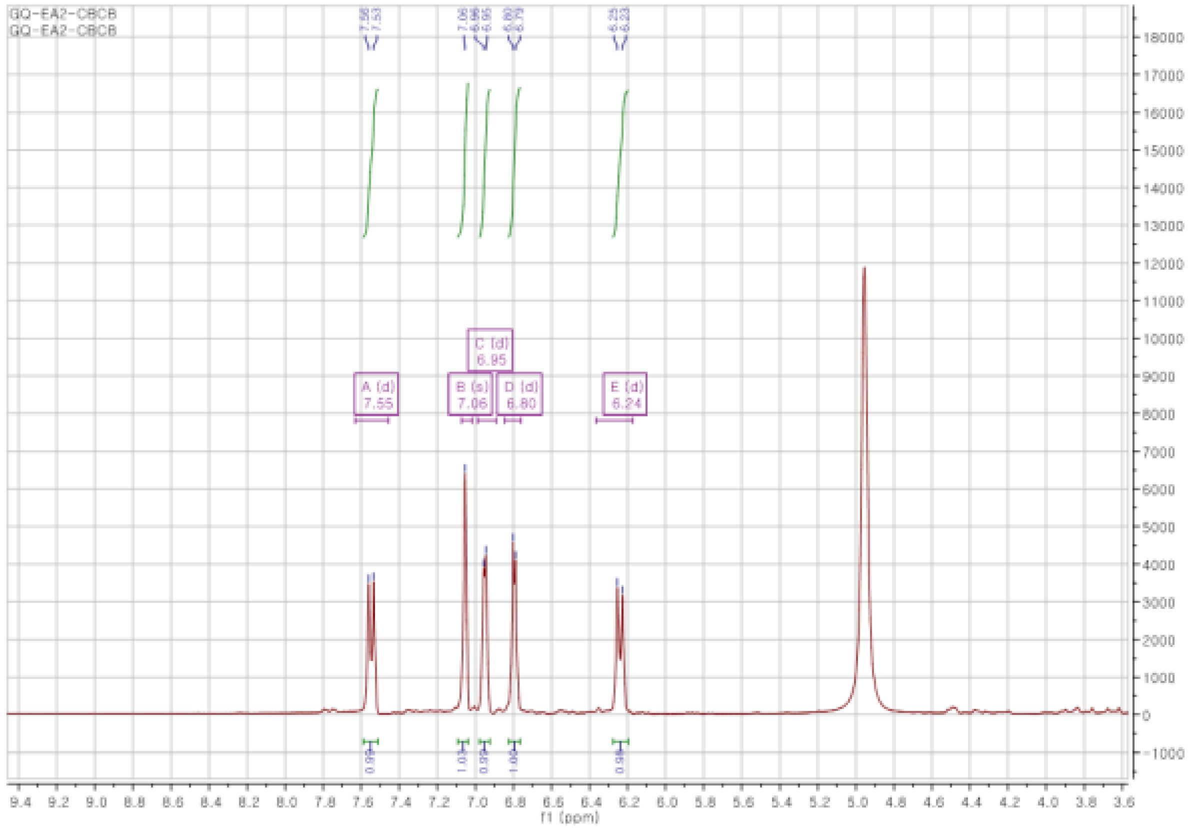

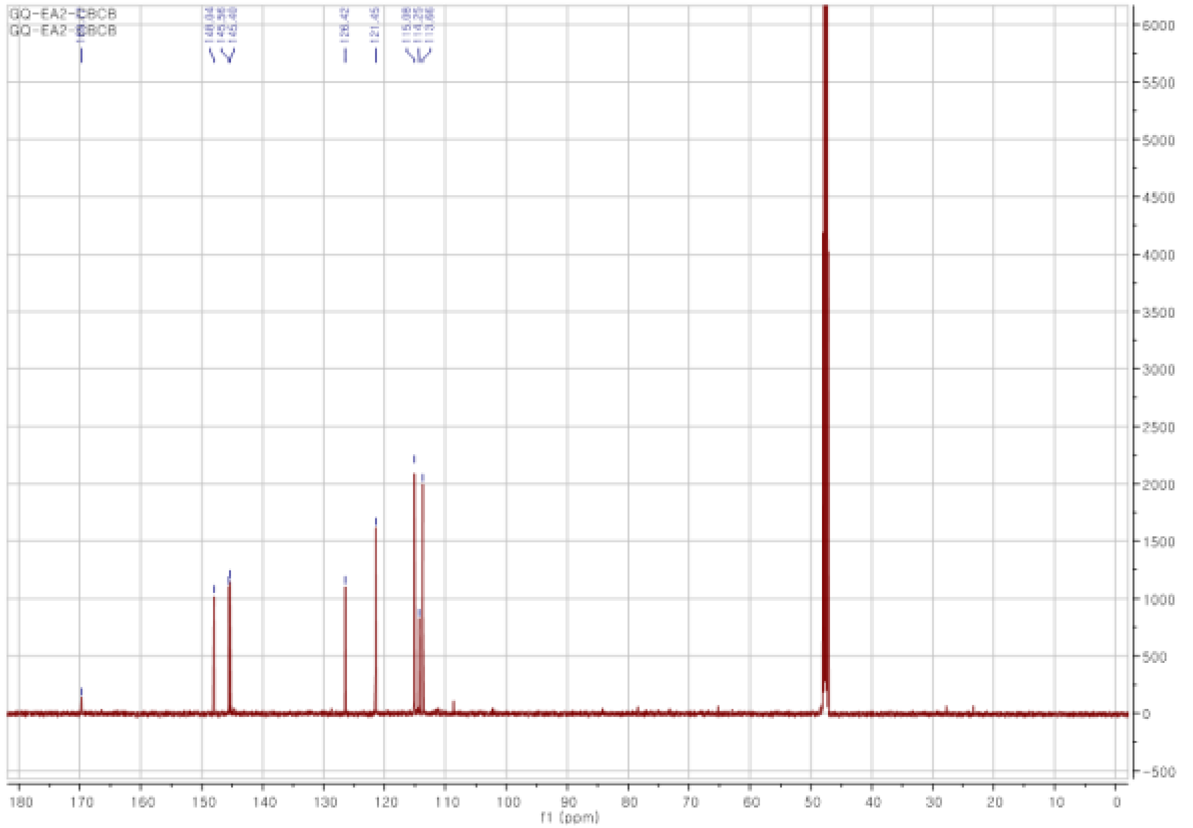

S20. ^{1}H and ^{13}C NMR spectrum of compound **19** in methanol-d_4 (600 and 150 MHz)

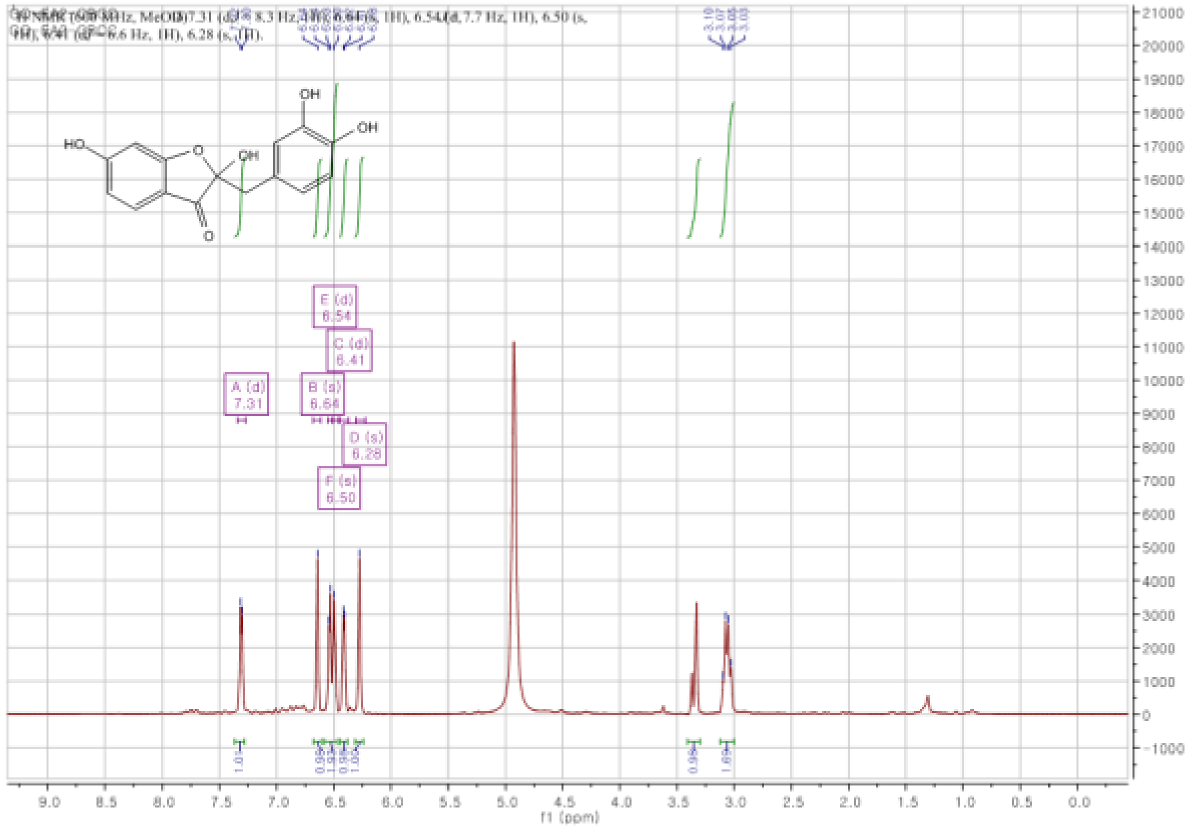

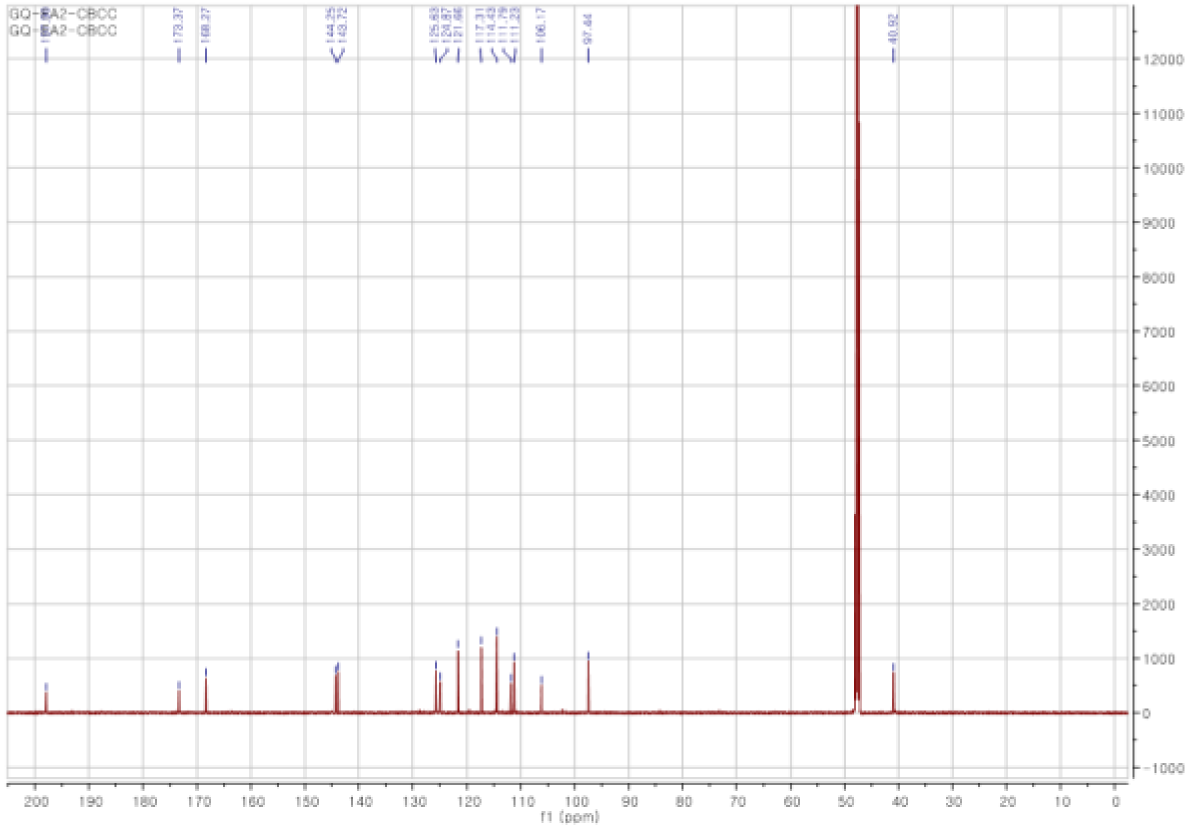

S21. ^{1}H and ^{13}C NMR spectrum of compound **20** in methanol-d_4 (600 and 150 MHz)

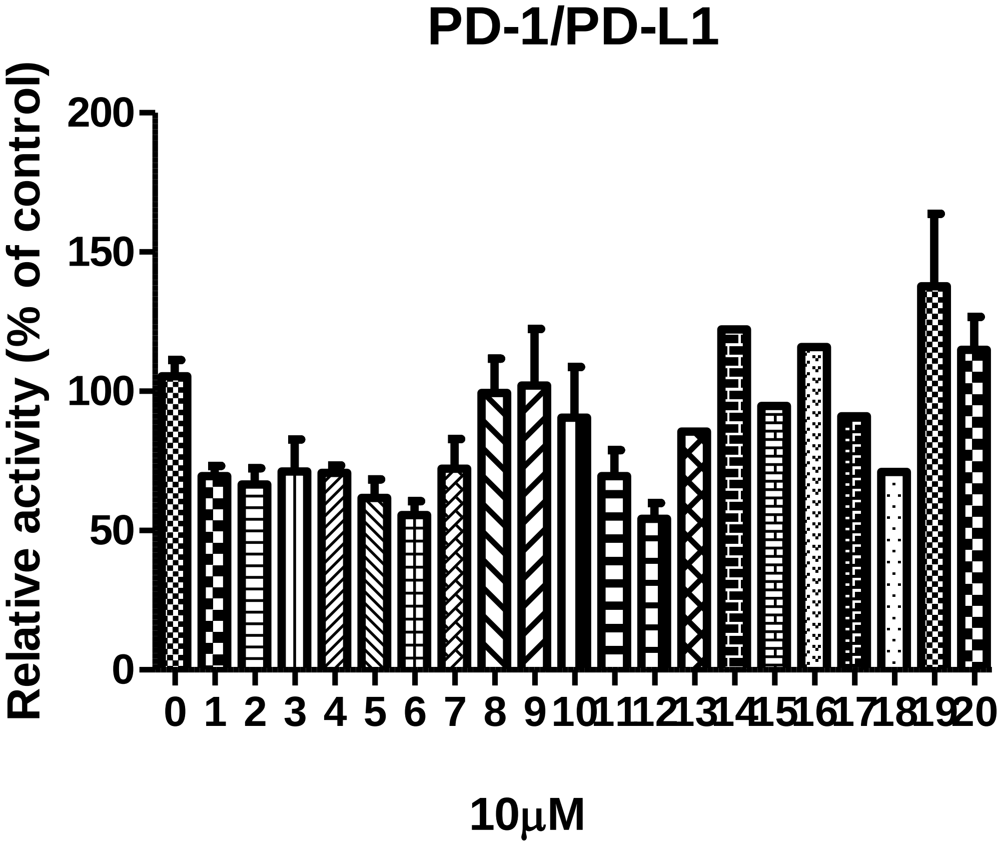

S22. Blocking effect of PD-1/PD-L1 interaction of isolated compound **1**–**20** at 10μM.

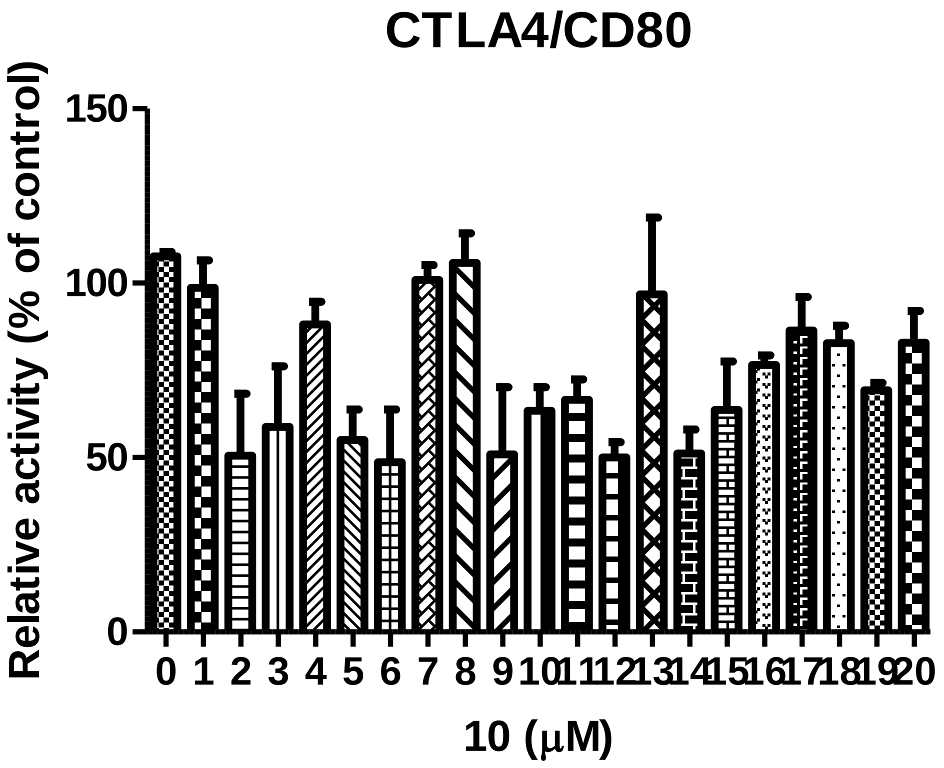

S23. Blocking effect of CTLA4/CD80 interaction of isolated compound **1**–**20** at 10μM.
